# 6-Methyl-5-hepten-2-one promotes programmed cell death during superficial scald development in pear

**DOI:** 10.1186/s43897-024-00107-1

**Published:** 2024-08-27

**Authors:** Junpeng Niu, Mingzhen Xu, Xu Zhang, Luqi Li, Weiqi Luo, Meng Ma, Lin Zhu, Decai Tian, Shaoling Zhang, Bing Xie, Guodong Wang, Libin Wang, Wei Hui

**Affiliations:** 1 College of Life Sciences, Shaanxi Normal University, Xi’an, 710119 China; 2 State Key Laboratory of Crop Genetics and Germplasm Enhancement, Nanjing Agricultural University, Nanjing, 210095 China; 3 Center for Integrated Pest Management, North Carolina State University, Raleigh, NC 27606 USA; 4 Engineering Research Center of High Value Utilization of Western China Fruit Resources, Ministry of Education, Xi’an, 710119 China

**Keywords:** Pear, Superficial scald, Cold storage, 6-Methyl-5-hepten-2-one, Programmed cell death

## Abstract

Plants possess the ability to induce programmed cell death (PCD) in response to abiotic and biotic stresses; nevertheless, the evidence on PCD initiation during pear scald development and the involvement of the scald trigger 6-methyl-5-hepten-2-one (MHO) in this process is rudimentary. *Pyrus bretschneideri* Rehd. cv. ‘Dangshansuli’ pear was used to validate such hypothesis. The results showed that superficial scald occurred after 120-d chilling exposure, which accompanied by typical PCD-associated morphological alterations, such as plasmolysis, cell shrinkage, cytosolic and nuclear condensation, vacuolar collapse, tonoplast disruption, subcellular organelle swelling, and DNA fragmentation. These symptoms were aggravated after MHO fumigation but alleviated by diphenylamine (DPA) dipping. Through transcriptome assay, 24 out of 146 PCD-related genes, which were transcribed during cold storage, were identified as the key candidate members responsible for these cellular biological alternations upon scald development. Among these, *PbrCNGC1*, *PbrGnai1*, *PbrACD6*, and *PbrSOBIR1* were implicated in the MHO signaling pathway. Additionally, PbrWRKY2, 34 and 39 could bind to the W-box element in the promoter of *PbrGnai1* or *PbrSOBIR1* and activate their transcription, as confirmed by dual-luciferase, yeast one-hybrid, and transient overexpression assays. Hence, our study confirms the PCD initiation during scald development and explores the critical role of MHO in this process.

**Supplementary Information:**

The online version contains supplementary material available at 10.1186/s43897-024-00107-1.

## Core

MHO plays a positive role and further promotes PCD during superficial scald development in pear fruit. *PbrCNGC1*, *PbrGnai1*, *PbrACD6*, and *PbrSOBIR1* function through the MHO signaling pathway. In addition, PbrWRKY2, 34, and 39 is the upstream regulators of *PbrGnai1* or *PbrSOBIR1*.

## Gene and accession numbers

Sequence data from this article can be found in the database of the pear genome database (http://peargenome.njau.edu.cn/) under the accession numbers: *PbrCNGC1* (*Pbr013608.1*), *PbrGnai1* (*Pbr029287.1*), *PbrACD6* (*Pbr027364.1*), *PbrSOBIR1* (*Pbr024019.1*), *PbrMYB7* (*Pbr009229.1*), *PbrbZIP36* (*Pbr015119.3*), *PbrWRKY2* (*Pbr018725.1*), *PbrWRKY34* (*Pbr019030.1*), *PbrWRKY39* (*Pbr029646.1*), *PbrWRKY88* (*Pbr015939.1*).

## Introduction

Superficial scald is a physiological disorder of pear and apple, which occurs during or after cold storage (Giné-Bordonaba et al. 2020; Qian et al. 2021; Zhang et al. 2023). Over the past half-century, various physio-chemical treatments have been explored to effectively control its development, including diphenylamine (DPA) dipping, 1-methylcyclopropene (1-MCP) fumigation, and controlled atmosphere (CA) storage (Hui et al. 2016; Dias et al. 2020; Qian et al. 2021). For example, 2.0 g L^-1^ DPA dipping for 60 s or 1.0 μL L^-1^ 1-MCP fumigation for 24 h has shown the potential to mitigate scald development when ‘Dangshansuli’ fruit was exposed to cold storage (Hui et al. 2016). Similar results have been observed in ‘Granny Smith’ apple during CA storage (3 % CO_2_ & 2 % O_2_) (Erkan et al. 2004). Although these handling practices demonstrates the potential in inhibiting scald development in pear and apple of certain cultivars, they could not completely resolve the problem (Gago et al. 2015; Lindo-García et al. 2021). Thus, it is urgent to investigate the underlying mechanism of such disorder.

Superficial scald has been proposed to be the result of an imbalance between the chilling-induced oxidant and antioxidant (Hui et al. 2016; Qian et al. 2021; Zhang et al. 2024). Chilling exposure would impair the cytochrome pathway of electron transport, leading to the accumulation of reactive oxygen species (ROS), which facilitates the α-farnesene oxidation into the scald trigger, 6-methyl-5-hepten-2-one (MHO) (Rowan et al. 1995 and 2001; Whitaker and Saftner 2000; Gong et al. 2021; Zhang et al. 2023; Vittani et al. 2023). Consistent with this, superoxide anion (O_2_·^¯^) and hydrogen peroxide (H_2_O_2_), conjugated trienes, and conjugated trienols accumulate in pear fruit with the prolonged low-temperature storage, resulting in an MHO burst when scald takes place (Sabban-Amin et al. 2011; Feng et al. 2018). Moreover, exogenous MHO fumigation triggers scald development, whereas the MHO inhibitor, DPA, plays a negative role in this process (Hou et al. 2013; Hui et al. 2016).

Programmed cell death (PCD) is a genetically regulated cellular process that functions in plant growth, development and adaptation to abiotic and biotic stresses (da Hora Junior et al. 2012; Fendrych et al. 2014; Petrov et al. 2015; Van Aken and Van Breusegem 2015; Zhou et al. 2018; Zheng et al. 2019; Park et al. 2023). ROS such as O_2_·^¯^, H_2_O_2_, and hydroxyl free radical (‧OH), along with several other metabolites like nitric oxide (NO) and adenosine triphosphate (ATP), are proposedly involved in plant PCD signaling (Van Aken and Van Breusegem 2015). Notably, once PCD is initiated, various morphological alternations take place in the cells, such as plasmolysis, cell shrinkage, plasma membrane rupture, cytosolic and nuclear condensation, vacuolar collapse, tonoplast disruption, subcellular organelle swelling, DNA fragmentation, and cytochrome c (Cyt c) release (Latrasse et al. 2016; Bedoui et al. 2020).

Proteins serve as the primary executor of vital life processes (Sun and Xu 2010). To date, numerous proteins involved in the PCD process have been identified from plants (Petrov et al. 2015). In *O. sativa* anthers, Argonaute 2 controls the tapetal PCD initiation through epigenetically regulating *Hexokinase 1* expression (Zheng et al. 2019). Heat Shock Protein 70, located in the mitochondria of *O. sativa*, has a negative impact on the activation of the PCD process during temperature elevation or H_2_O_2_ treatment (Qi et al. 2011). Similarly, *Arabidopsis* Metacaspase-8 modulates ultraviolet light- or H_2_O_2_-induced PCD process (He et al. 2008).

Superficial scald is implicated to be linked with the PCD process based on the expression profiles of several PCD-related genes during the cold storage of apple fruit, including *Defender Against Cell Death 1* (*DAD1*), *Defense, No Death 1* (*DND1*), and *Lesion Simulating Disease 1* (*LSD1*) (Busatto et al. 2014; Du et al. 2017; Ding et al. 2019). Nevertheless, our understanding of PCD activation during pear scald development and the role of MHO in this process are still limited. In this study, a comprehensive analysis, including quality assessment, physio-chemical measurement, cellular biological investigation, and several molecular biological technologies, was conducted to confirm PCD occurrence during scald development in pear fruit and uncover the role of MHO in this process.

## Results

### Dynamic changes of quality parameters

Superficial scald occurred after 120-d cold storage and progressively expanded to almost all fruit (Fig. S1). As shown in Fig. S1, scald incidence increased from 0.00 % on day 0 and 60, to 6.94 % on day 120, and further to 24.67 % on day 180; and scald index increased from 0.00 % on day 0 and 60, to 4.94 % and 18.33 % on day 120 and 180, respectively. Intriguingly, exogenous DPA dipping delayed the scald occurrence until day 180, which was associated with the inhibited scald incidence (6.29 %) and index (4.90 %) (Fig. S1). Conversely, MHO fumigation accelerated scald occurrence, manifesting as early as day 60, and intensified scald symptoms during cold storage (Fig. S1). On day 180, the scald incidence and index of the MHO-treated fruit were 96.67 % and 65.3 %, respectively (Fig. S1). However, no difference was observed for firmness, total soluble solid (TSS), and titratable acids (TA) in sarcocarp tissues of different treatments at the same sampling time (Fig. S2).

### Dynamic changes of the physio-biochemical parameters

**Fig. 1:**
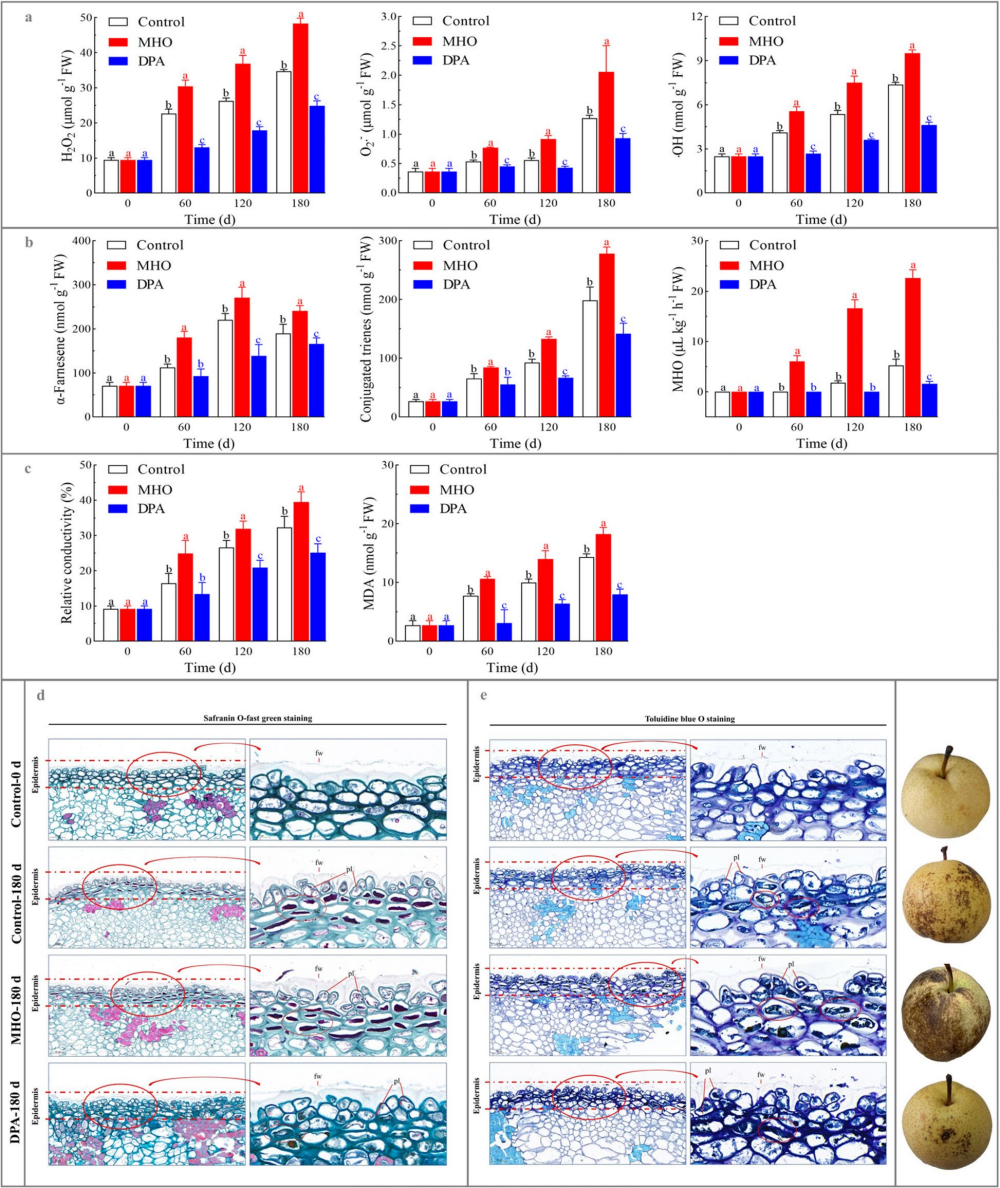
Dynamic changes of the physio-biochemical attributes, safranin O-fast green staining, and toluidine blue O staining during cold storage of pear fruits. **a** ROS-related metabolites (H_2_O_2_, O_2_·¯, and ‧OH); **b** α-farnesene-related metabolites (α-farnesene, conjugated trienes, and MHO); **c** relative conductivity and MDA. ‘Dangshansuli’ fruits were randomly divided into three treatments: H_2_O dipping (control), MHO fumigation, and DPA dipping. The samples were collected every 60 d followed by a 7-d shelf life at 20 ℃. The data are the mean values ± SD of three biological replicates. Vertical bars labeled with the same letter indicate no significant difference between samples at the same sampling time at *P* < 0.05. **d** the safranin O-fast green staining images; **e** the toluidine blue O staining images. ‘Dangshansuli’ fruits were randomly divided into three treatments: H_2_O dipping (control), MHO fumigation, and DPA dipping. The samples were collected every 60 d followed by a 7-d shelf life at 20 ℃. Abbreviations: fw, fruit wax; pl, plasmolysis; sc, shrunken cytoplasm

H_2_O_2_, O_2_·^¯^, ‧OH, α-farnesene, conjugated trienes, MDA, and relative conductivity gradually accumulated in the control. Specifically, their levels increased from 9.47 µmol g^-1^ FW, 0.36 µmol g^-1^ FW, 2.50 nmol g^-1^ FW, 70.61 nmol g^-1^ FW, 26.62 nmol g^-1^ FW, 2.74 nmol g^-1^ FW, and 9.18 %, respectively, on day 0, to 34.74 µmol g^-1^ FW, 1.27 µmol g^-1^ FW, 7.38 nmol g^-1^ FW, 189.50 nmol g^-1^ FW, 198.53 nmol g^-1^ FW, 14.33 nmol g^-1^ FW, and 32.31 %, respectively, on day 180 (Fig. 1a-c). MHO could not be detected until day 120 when scald symptom appeared (Fig. 1b).

Exogenous MHO fumigation promoted the accumulation of H_2_O_2_, O_2_·^¯^, ‧OH, α-farnesene, conjugated trienes, relative conductivity, and MDA, with an advanced MHO burst (6.08 μL kg^-1^ h^-1^ FW) on day 60 (Fig. 1a-c). In contrast, the DPA-treated fruit exhibited a delayed MHO burst, which took place on day 180 (Fig. 1a-c).

Furthermore, the extremely strong positive correlations were observed among scald incidence/index, ROS-related metabolites (H_2_O_2_, O_2_·^¯^, and ‧OH), α-farnesene-related metabolites (α-farnesene, conjugated trienes, and MHO), relative conductivity, and MDA during cold storage of ‘Dangshansuli’ fruit (correlation coefficient > 0.8, Fig. S3).

### Cellular biological alternations

As ROS is implicated to participate in plant PCD signaling (Van Aken and Van Breusegem 2015), then further experiment was conducted to validate the occurrence of the PCD-related morphological alternations during scald development as well as the role of MHO in this process with the aid of cellular biological techniques, including safranin O-fast green staining, toluidine blue O staining, TdT-mediated dUTP nick-end labeling (TUNEL), and transmission electron microscope (TEM) analysis.

**Fig. 2:**
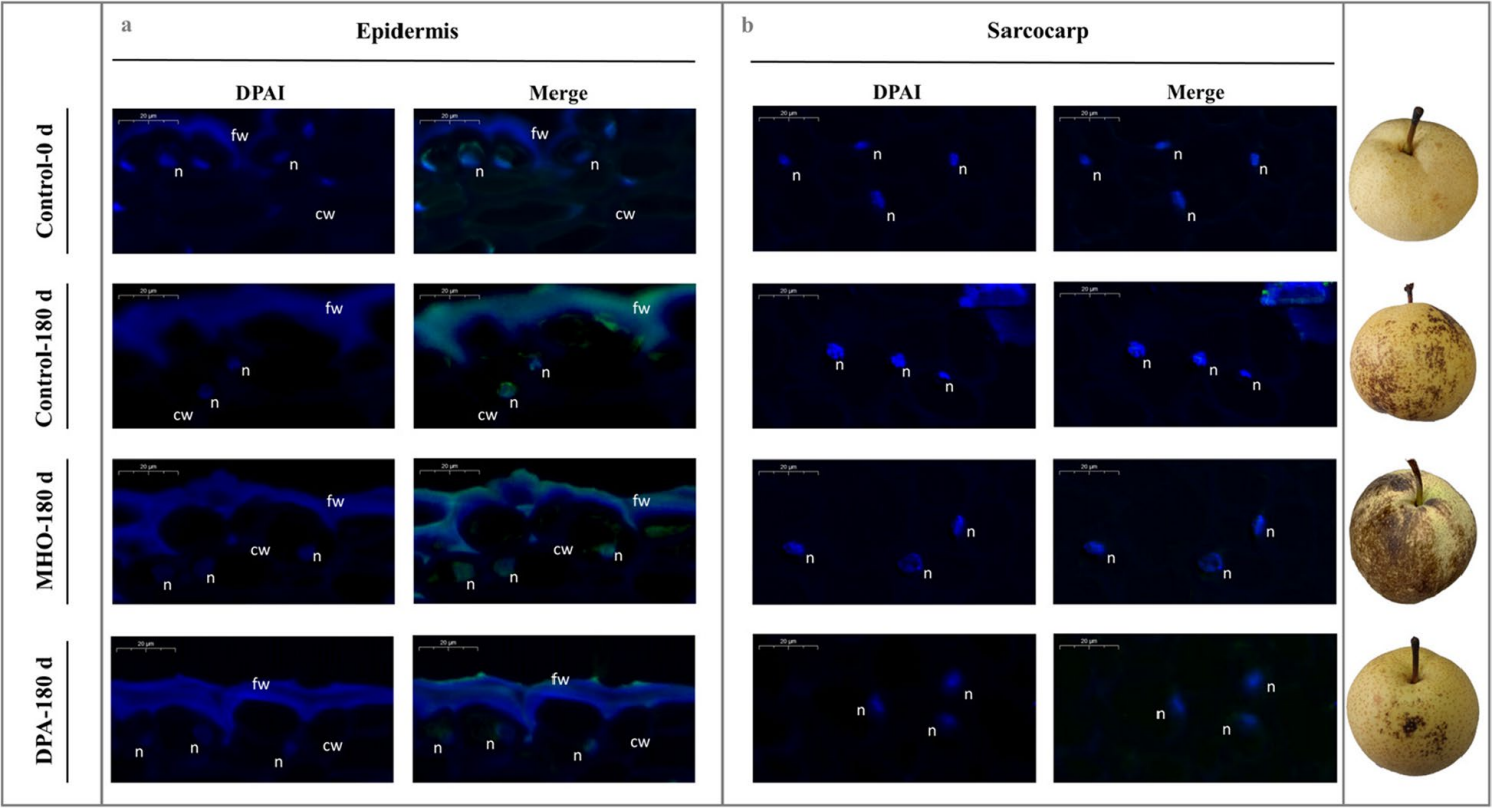
TUNEL staining of pear fruits. ‘Dangshansuli’ fruits were randomly divided into three treatments: H_2_O dipping (control), MHO fumigation, and DPA dipping. The samples were collected every 60 d followed by a 7-d shelf life at 20 ℃. Abbreviations: cw, cell wall; fw, fruit wax; n, DNA

As shown in Figs. 1d-e, 2, and S4, the cell membrane and cell wall in the epidermis and sarcocarp of the control on day 0 were intact and tightly connected, without any PCD-related symptoms. After 180 d of chilling exposure, scald occurred in the epidermal cells along with the development of several typical PCD-related symptoms, such as plasmolysis, cell shrinkage, DNA fragmentation, cytosolic and nuclear condensation, vacuolar collapse, tonoplast disruption, and swelling of subcellular organelles, such as endoplasmic reticulum, chloroplasts, and mitochondria. Exogenous MHO fumigation exacerbated aforementioned morphological changes in the epidermal cells, while DPA dipping treatment alleviated these alterations. However, no typical PCD-related symptoms were observed in sarcocarp tissue on day 180.

Taken together, these results imply that the PCD-related morphological alternations take place during scald development, and MHO is likely to play a critical role in promoting this process.

### Identification of the PCD-related genes involved in scald development

**Fig. 3:**
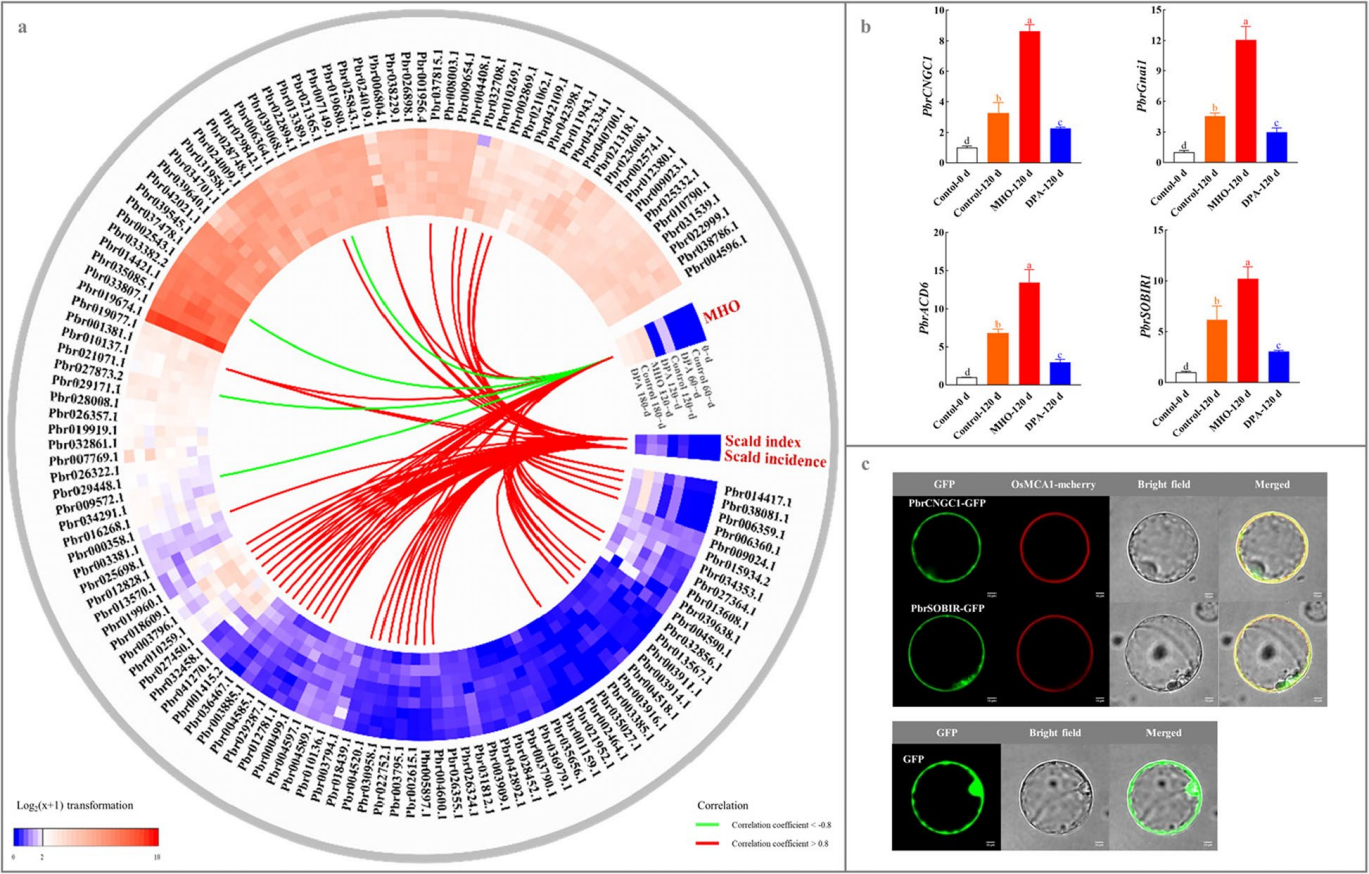
Identification of the PCD-related genes involved in scald development. **a** Expression profiles of 146 PCD-related genes and their correlation with scald incidence/index and MHO content. ‘Dangshansuli’ fruits were randomly divided into three treatments: H_2_O dipping (control), MHO fumigation, and DPA dipping. A total of 146 PCD-related genes are identified from pear genome based on transcriptome annotation (Table S2). Data, adapted from the transcriptome assay, present the mean values (Fragments Per Kilobase Million, FPKM) of three biological replicates. The color scale represents the normalized log2-transformed (FPKM + 1), where red indicates a high level, blue indicates a low level, and white indicates a medium level. Pearson correlations among attributes are visualized as a heatmap, with extremely strong negative correlations shown as green lines (correlation coefficients *<* -0.8) and positive correlations shown as red lines (correlation coefficients > 0.8). **b** qRT-PCR assay of the expression profiles of four PCD-related genes. The expression abundance of each gene in 0-d fruit was normalized to 1.0 for the qRT-PCR assay. The data are the mean values ± SD of three biological replicates. Vertical bars labeled with the same letter indicate no significant difference between samples at the same sampling time at *P <* 0.05. **c** The subcellular localization of PbrCNGC1 and PbrSOBIR1. OsMCA1-mcherry is used as the plasma membrane marker (Kurusu et al. 2012)

Proteins, which are encoded by the correspondent genes, are the primary executors in plant response to abiotic and biotic stresses (Chakravarthy et al. 2003; Sun and Xu 2010; Liu et al. 2015). Thus, we performed transcriptome assays to identify the PCD-related genes responsible for the cellular biological alternations as mentioned above (Experiment II). As shown in Fig. 3a and Table S2, a total of 146 PCD-related genes were transcribed during cold storage, exhibiting the diverse expression patterns. Among them, 24 members demonstrated a strong correlation with scald incidence/index (absolute correlation coefficient > 0.8), implying that they are responsible for the morphological changes during scald development.

Moreover, the expression levels of four genes, including *Pbr013608.1*, *Pbr029287.1*, *Pbr027364.1*, and *Pbr024019.1*, which displayed the extremely strong positive correlations with MHO level, were significantly upregulated by MHO fumigation but downregulated by DPA dipping (fold change ≥ 1.5 and false discovery rate (FDR) < 0.05; correlation coefficient > 0.8; Fig. 3a). Quantitative real-time polymerase chain reaction (qRT-PCR) assay validated their expression patterns (Fig. 3b). Taken together, these results imply that these four members might be involved in MHO signaling pathway.

Based on transcriptome annotation (Table S2), *Pbr013608.1* encoded a Cyclic Nucleotide-gated Ion Channel 1-Like Protein (named as PbrCNGC1); *Pbr029287.1* encoded a Guanine Nucleotide-binding Protein Alpha-1 Subunit (named as PbrGnai1); *Pbr027364.1* encoded an Accelerated Cell Death 6-Like Protein (named as PbrACD6); on the other hand, *Pbr024019.1* encoded a Leucine-Rich Repeat Receptor-like Serine/Threonine/Tyrosine-protein Kinase SOBIR1 (named as PbrSOBIR1).

Afterwards, we assayed their characteristics. Except for PbrACD6, their protein sequences exhibited high identity to the homologues in *O. sativa*, *Arabidopsis*, and *Nicotiana tabacum*, suggesting the functional conservation during plant evolution (Fig. S5) (Sunkar et al. 2000; Lu et al. 2003; Zhang et al. 2021). PbrCNGC1 and PbrACD6 possessed six and four transmembrane helices, respectively, while PbrSOBIR1 contained one transmembrane helix and a signal peptide in its N-terminus (Fig. S6). Their physio-biochemical features were summarized in Table S3. Except for PbrACD6, the other three were predicted to be located in the plasma membrane (Table S3). Subsequently, PbrCNGC1 and PbrSOBIR1 were randomly selected and confirmed the hypothesis (Fig. 3c and Table S3).

### Identification of the upstream regulators of *PbrCNGC1*, *PbrGnai1*, *PbrACD6* and *PbrSOBIR1*

**Fig. 4:**
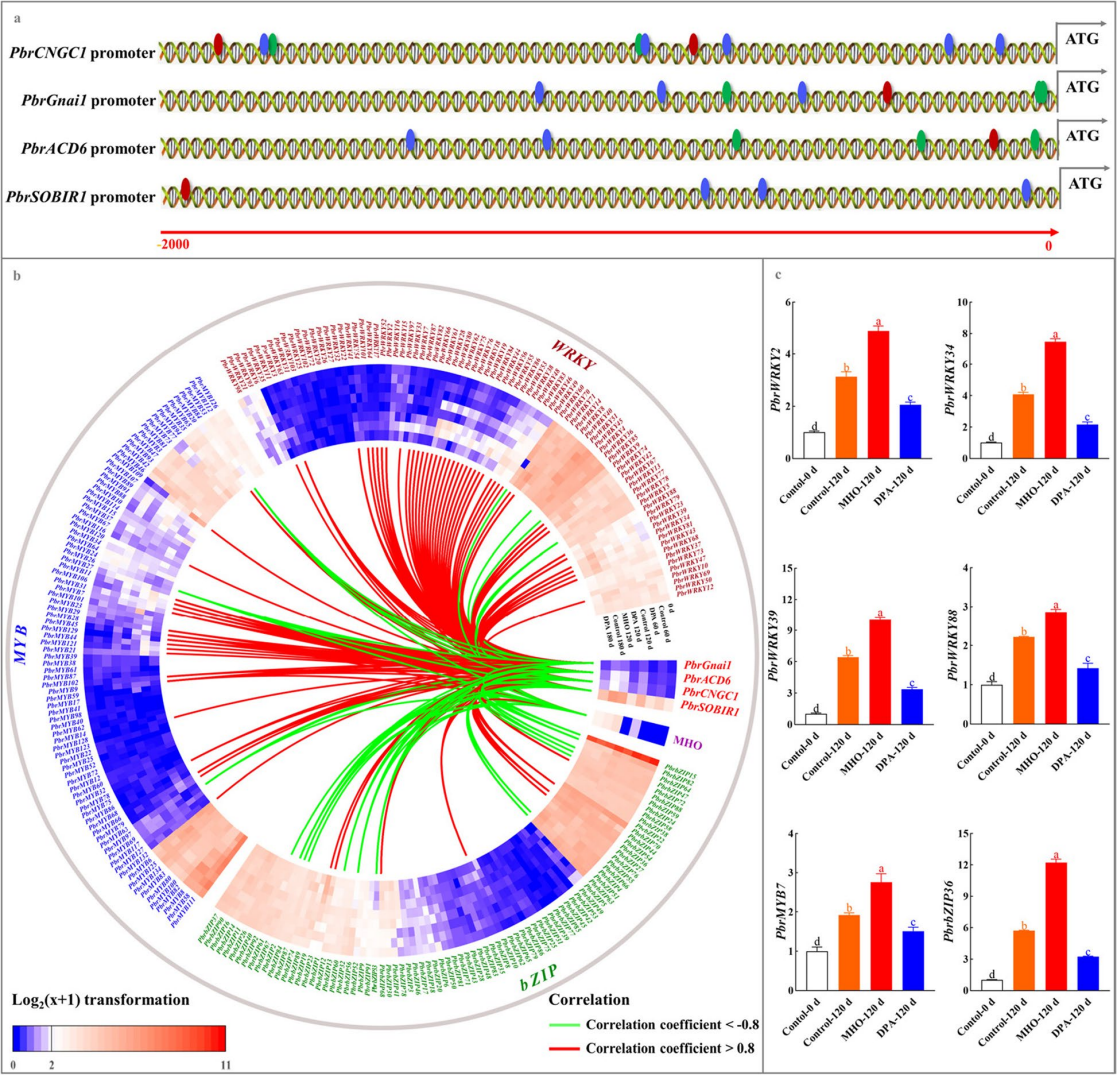
Identification of TFs possibly regulating *PbrCNGC1*, *PbrGnai1*, *PbrACD6* and *PbrSOBIR1* expression. **a** Schematic model of *cis*-acting element distributions in *PbrCNGC1*, *PbrGnai1*, *PbrACD6*, and *PbrSOBIR1* promoters. Red, green, and blue ellipses represent W-boxes, G-boxes, and MYB-binding sites, respectively. **b** Expression profiles of TFs during cold storage of pear fruits and their correlation with MHO level as well as mRNA abundances of four PCD-related genes. ‘Dangshansuli’ fruits were randomly divided into three treatments: H_2_O dipping (control), MHO fumigation, and DPA dipping. *PbrWRKYs*, *PbrbZIPs*, and *PbrMYBs* were characterized from the *P. bretschneideri* Rehd. genome (Huang et al. 2015; Cao et al. 2016; Ma et al. 2021). Data, adapted from the transcriptome assay, present the mean values (FPKM) of three biological replicates. The color scale represents the normalized log2-transformed values (FPKM + 1), with red indicating a high level, blue indicating a low level, and white indicating a medium level. Pearson correlations among attributes are visualized as a heatmap, with extremely strong negative correlations (correlation coefficients *<* -0.8) represented by green lines and positive correlations (correlation coefficients > 0.8) represented by red lines. **c** qRT-PCR assay of the expression profiles of *PbrWRKY2, 34, 39 and 88*, *PbrMYB7*, and *PbrbZIP36*. The expression abundance of each gene in 0-d fruit was normalized to 1.0 for the qRT-PCR assay. The data are the mean values ± SD of three biological replicates. Vertical bars labeled with the same letter indicate no significant difference between samples at the same sampling time at *P <* 0.05

In plants, transcription factors (TFs) regulated the transcription of the downstream structural gene via binding to the correspondent *cis*-acting elements in its promoter (Chakravarthy et al. 2003). WRKYs, MYBs, and bZIPs are the most common TFs involved in plant responses to abiotic and biotic stresses (Chakravarthy et al. 2003; Liu et al. 2015). With the aid of the PlantCARE database, a bunch of W-box elements, G-box elements, and MYB-binding sites were identified from the promoters of *PbrCNGC1*, *PbrGnai1*, *PbrACD6*, and *PbrSOBIR1* (Fig. 4a and Table S4). Thus, we investigated the expression profiles of the related TFs during the cold storage of ‘Dangshansuli’ fruit (Experiment II).

As shown in Fig. 4a-b and Tables S4-S6, the expression levels of 23 members, which were upregulated by MHO but downregulated by DPA (fold change ≥ 1.5 and FDR < 0.05), exhibited the extremely strong positive correlations with MHO level and the mRNA abundances of *PbrCNGC1*, *PbrGnai1*, *PbrACD6*, and *PbrSOBIR1* (correlation coefficient > 0.8). qRT-PCR assay validated the expression patterns of several members (Fig. 4b, c). By the PlantRegMap database, their possible binding sites in the promoters of *PbrCNGC1*, *PbrGnai1*, *PbrACD6*, or *PbrSOBIR1* were identified and then summarized in Table S6. Taken together, these results suggest that these 23 TFs are the possible positive upstream regulators of *PbrCNGC1*, *PbrGnai1*, *PbrACD6*, and *PbrSOBIR1*, and are involved in MHO signaling pathway as well.

### PbrWRKY2, 34 and 39 could bind to *PbrGnai1* or *PbrSOBIR1* promoter and then activate their expression

**Fig. 5:**
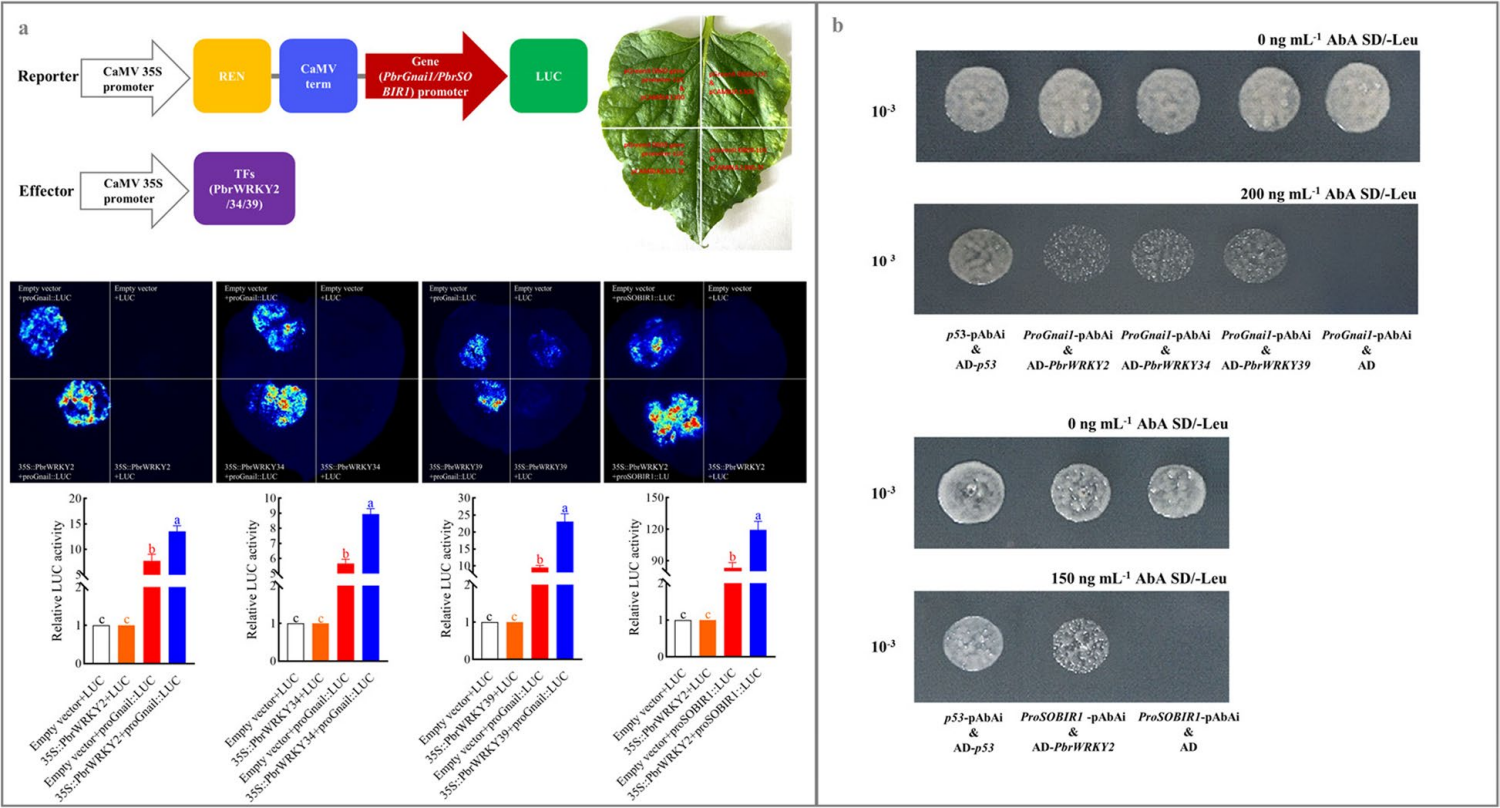
PbrWRKY2, 34 and 39 could bind to *PbrGnai1* or *PbrSOBIR1* promoters and activate their expression. **a** Dual-luciferase (dual-LUC) assay. Co-transformants containing pCAMBIA1300 & pGreen 0800-LUC vectors, pCAMBIA1300 & pGreen 0800-*PbrCNGC1pro*/*PbrGnai1pro*/*PbrSOBIR1pro*-LUC vectors, or pCAMBIA1300-*PbrWRKY2/34/39* & pGreen 0800-LUC vectors were used as control. The data are the mean values ± SD of three biological replicates, and vertical bars labelled labeled with the same letter indicate no significant difference between samples at *P <* 0.05. **b** Y1H assay. Yeast cell co-transformed with AD-*p53* & *p53*-AbAi was used as the positive control, while yeasts co-transformed with the empty AD vector and each bait as the negative controls

Due to their relatively high coefficients with four PCD-related genes during scald development, PbrWRKY2, 34 and 39 were selected for further study (Fig. 4a). When compared with the control, a considerable increment of the relative luciferase (LUC) activity was observed in *N. benthamiana* leaves co-transformed with PbrWRKY2/34/39 & *PbrGnai1* promoter or PbrWRKY2 & *PbrSOBIR1* promoter; however, such increment disappeared after the mutation of W-box elements in their promoters (Fig. 5a and S7-S8). However, such increment disappeared after the mutation of W-box elements in their promoters (Fig. S8a). Based on the result of yeast one-hybrid (Y1H) assay, PbrWRKY2, 34, and 39 could bind to the W-box element in *PbrGnai1* promoter, while PbrWRKY2 specifically bound to the W-box element in *PbrSOBIR1* promoter (Fig. 5b); on the other hand, such interaction vanished after the mutation of W-box elements in their promoters (Fig. S8b).

**Fig. 6:**
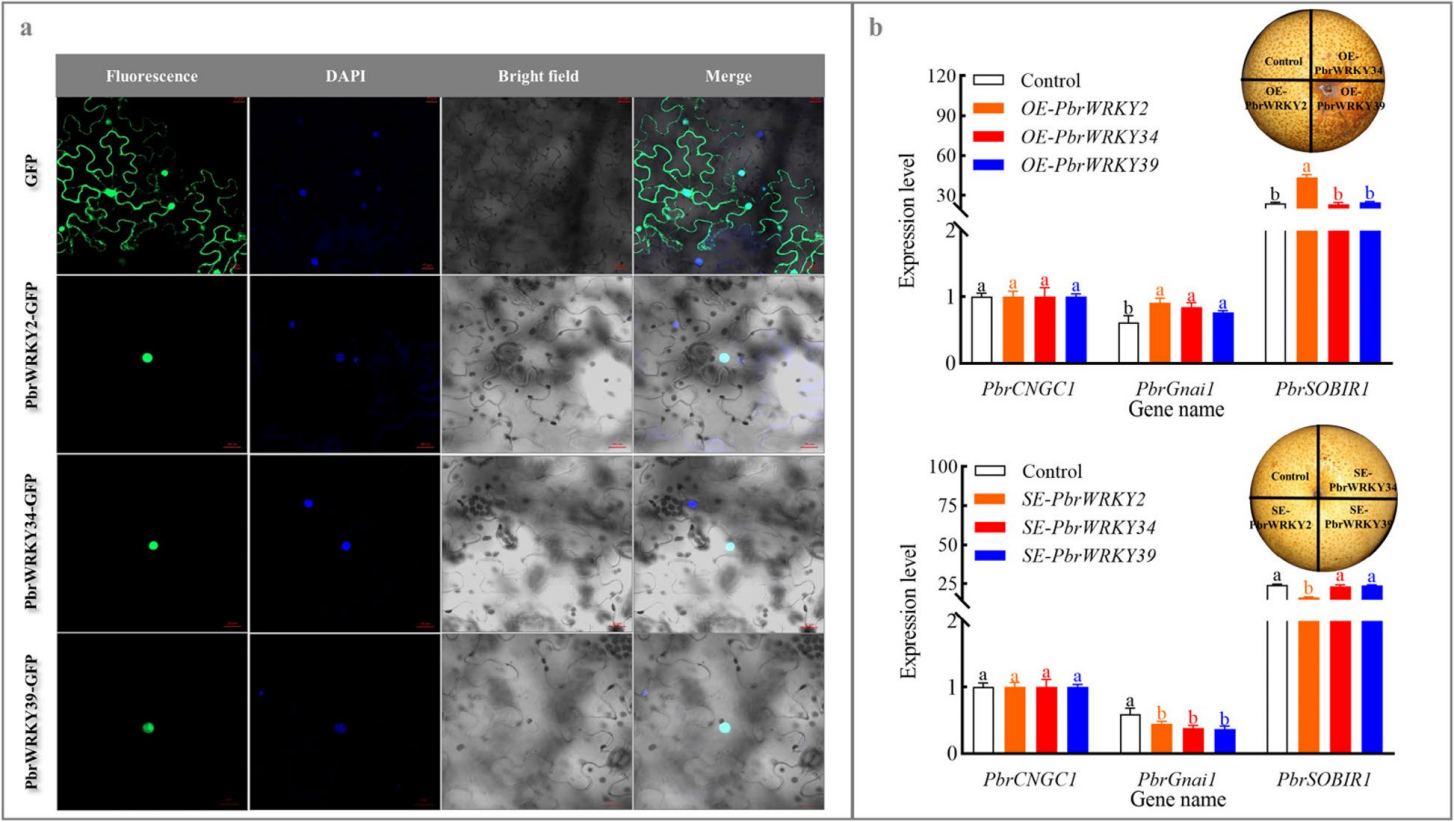
Functional validation of PbrWRKY2, 34 and 39 in regulating *PbrGnai1* or *PbrSOBIR1* expression in the epidermal tissue of pear fruit. **a** Subcellular localizations of PbrWRKY2, 34 and 39. And 4’, 6-diamidino-2-phenylindole (DAPI) was used as a nuclear indicator. **b** The impact of transient transformation of pear fruit on *PbrGnai1* and *PbrSOBIR1* expression. Fruits transformed with the empty pCAMBIA1300 vector served as a control for *PbrWRKY2/34/39*-overexpressing fruit, while fruits co-transformed with empty TRV1 and TRV2 served as a control for *PbrWRKY2/34/39*-silenced fruit. The expression abundance of *PbrCNGC1* in control fruit was set as 1.0 for the qRT-PCR assay. The data are the mean values ± SD of three biological replicates. Vertical bars labeled with the same letter indicate no significant difference between samples at the same sampling time at *P <* 0.05

Subsequently, we assayed their function in pear fruit. All three TFs were located in the nuclei of *N. benthamiana* leaf (Fig. 6a). Transient overexpression of *PbrWRKY2*, *34* and *39* in the epidermis of ‘Dangshansuli’ fruit upregulated *PbrGnai1* expression; meanwhile, a higher abundance of *PbrSOBIR1* mRNA was detected in the *PbrWRKY2*-overexpressing pear than the control (Fig. 6b and S9). Conversely, the transient silence of *PbrWRKY2*, *34* and *39* led to the suppressed *PbrGnai1* or *PbrSOBIR1* transcription (Fig. 6b and S9).

Taken together, our results confirm that PbrWRKY2, 34, and 39 could bind to the W-box element in *PbrGnai1* or *PbrSOBIR1* promoter, thereby activating their expression.

### Impact of MHO fumigation concentration and time on scald development and *PbrWRKY2*, *34* and *39* expression levels

As the activation or suppression of gene expression is transcriptionally regulated by several TFs, overexpression or mutation of only one or two TFs might not absolutely change the phenotype (Prelich 2012; Strader et al. 2022; He et al. 2023). In this study, 23 TFs were likely positive upstream regulators of *PbrCNGC1*, *PbrGnai1*, *PbrACD6*, and *PbrSOBIR1* (Fig. 4). Thus, we investigated the relationship among exogenous MHO fumigation concentration (Experiment III) and time (Experiment IV), *PbrWRKYs* expression levels, and scald incidence/index to determine whether MHO could directly activate PCD process or activate it through these three WRKY TFs. As shown in Fig. S10, a considerable elevation of *PbrWRKY2*, *34* and *39* transcription was detected after 0.25 or 1.00 mL L^-1^ MHO fumigation for 48 h, which was associated with the occurrence of scald symptom; on the other hand, negligible increment of *PbrWRKYs* expression abundances as well as no evident scald symptom were observed in fruit treated with 0.00 or 0.01 mL L^-1^ MHO for 48 h (Fig. S10). Furthermore, 0.25 mL L^-1^ MHO fumigation for a short period (0-12 h) did not trigger the upregulation of *PbrWRKY2*, *34* and *39* expression and the occurrence of scald symptom; however, after 24-h fumigation, a considerable increment of *PbrWRKYs* transcription was detected in fruit harboring scald symptom (Fig. S11). In combination with the alternation of MHO abundance and *PbrWRKY2*, *34* and *39* expression profiles during the storage of ‘Dangshansuli’ fruit with/without chemical treatments (Fig. 1 and 4), our results implied that MHO might activate PCD process partly through these three WRKY TFs.

## Discussion

Low-temperature storage is commonly applied to extend the postharvest marketing time of apple and pear (Rodrigues et al. 2024). However, such handling practice would lead to the onset of a chilling injury disorder, known as superficial scald in the epidermis of the fruits (Giné-Bordonaba et al. 2020; Qian et al. 2021; Vittani et al. 2023). Such disorder is considered to the consequence of the chilling-induced antioxidant imbalance (Lurie and Watkins 2012; Zhang et al. 2024). In agreement with the results of previous studies (Rowan et al. 1995 and 2001; Whitaker and Saftner 2000; Lurie and Watkins 2012; Gong et al. 2021), chilling exposure of ‘Dangshansuli’ fruit caused the accumulation of O_2_·^¯^, H_2_O_2_, and ‧OH, in association with a burst of the scald trigger, MHO (Fig. 1a, b); and the changes and correlation of these parameters implicated the roles of lipid peroxidation, ROS and α-farnesene metabolisms in scald development (Fig. 1a-c). Moreover, exogenous MHO fumigation triggered and promoted scald development in ‘Dangshansuli’ fruit, while DPA dipping showed the opposite effect (Fig. S1). Similar phenomena have been reported by Hui et al. (2016), using ‘Dangshansuli’ fruit as material.

PCD is a developmental or defensive process, which is well characterized in plants and animals (Rahikainen 2024). Although the triggering signaling mechanisms varies from species to species, PCD illustrates common features (Daneva et al. 2016; Elena-Real et al. 2021; Kaźmierczak et al. 2023). In this study, in accompany with the elevation of lipid peroxidation, the loss of cell membrane integrity as well as the accumulation of ROS and α-farnesene autoxidation, several typical PCD-related morphological changes took place in the epidermal cells after 180-d of cold storage (Figs. 1 and 2 and S4). These symptoms have also been detected in other horticultural fruits after low-temperature treatments (Kratsch and Wise 2000; Liang et al. 2022; Ramírez-Sánchez et al. 2022). For example, low-temperature storage of banana at the pre-climacteric stage caused DNA degradation or tailing (Ramírez-Sánchez et al. 2022). Likely, several typical PCD-related morphological changes, including chromatin condensation, DNA ladder, and Cyt c release, have been observed in the chilled pumpkins (Liang et al. 2022). Moreover, consistent with the results in apple fruit (Busatto et al. 2014; Du et al. 2017; Ding et al. 2019; Zhang et al. 2023), 24 PCD-related genes, whose expression altered with the prolonged chilling exposure, were proposedly responsible for these cellular biological changes upon scald development in ‘Dangshansuli’ fruit (Fig. 3a and Table S2). Therefore, our results implicate the occurrence of the PCD process during scald development.

It is generally believed that PCD is an active extinction process, which takes place after cells receive a certain signal or are stimulated by certain factors in order to maintain the stability of the internal environment (Liu et al. 2022a). ROS, NO, and ATP are the common signaling molecules involved in plant PCD process (Van Aken and Van Breusegem 2015). However, our knowledge on the role of MHO in plant PCD signaling is still rudimentary. In this study, by using exogenous MHO and DPA treatments, we explored that the occurrence of several typical PCD-related morphological changes as well as the expression of four PCD-related genes, including *PbrCNGC1*, *PbrGnai1*, *PbrACD6*, and *PbrSOBIR1*, were under the control of MHO (Figs. 1, 2 and 3, S4-S6, and Tables S2-S3). The functions of the homologous genes from other plants have been previously validated. *Arabidopsis* CNGC1, located in the plasma membrane, functions in Ca^2+^ uptake and triggers PCD process (Demidchik and Maathuis 2007). *ACD6* promotes salicylate accumulation, causing spontaneous cell death in *Arabidopsis* (Lu et al. 2003). The plasma membrane-located Gnai1 from *O. sativa* plays a critical role in epidermal cell death (Steffens and Sauter 2009). On the other hand, *SOBIR1* is involved in the elevated cell death of *Arabidopsis* (Gao et al. 2009). Therefore, these results imply that MHO might act as the signaling molecule of the PCD process during scald development in pear, and these four genes might be involved in MHO signaling pathway.

In this study, although postharvest MHO and DPA treatments influenced scald development in the epidermis of ‘Dangshansuli’ fruit, no significant differences in firmness, TSS, and TA in the sarcocarp were detected among samples (Fig. S2). Similar result was also observed between the scalded and unscalded ‘Chili’ pear (He et al. 2022) or between the control and 1-MCP-treated ‘Yali’ fruit upon scald development (Li et al. 2022). These results suggest that exogenous MHO and DPA treatments (concentration and treatment time), which were applied in this study, just influence the cellular metabolism of the epidermal tissue (Lurie and Watkins 2012).

In plants, the expression of the downstream structural gene was under the control of TFs through the interaction with the related *cis*-acting elements in its promoter (Chakravarthy et al. 2003). *Arabidopsis* NAC4 could bind to the promoters of *late upregulated in response to Hyaloperonospora parasitica 1* (*LURP1*), *WRKY40*, and *WRKY54*, promoting the pathogen-induced PCD process (Lee et al. 2017). Similarly, WSR1 from rapeseed, which could be phosphorylated by Ca^2+^-dependent protein kinase (CPK), modulates cell death and leaf senescence by regulating the expression of ROS-metabolism-related and salicylate-synthesis-related genes (Cui et al. 2020). In the present study, three out of 23 TFs proposedly involved in MHO signaling pathway, including PbrWRKY2, 34, and 39, were selected and validated to interact with the W-box element in the promoter of *PbrGnai1* or *PbrSOBIR1*, and then trigger their transcription (Figs. 4, 5 and 6, S7-S9, and Tables S4-S6). Taking account of the alternation of MHO level and *PbrWRKY2*, *34* and *39* expression profiles during the storage of ‘Dangshansuli’ fruit (Figs. 1 and 4) as well as the impact of MHO fumigation concentration and time on their expression profiles and thus scald development (Fig. S10-S11), our results imply that MHO might activate PCD process partly through these three WRKY TFs.

**Fig. 7:**
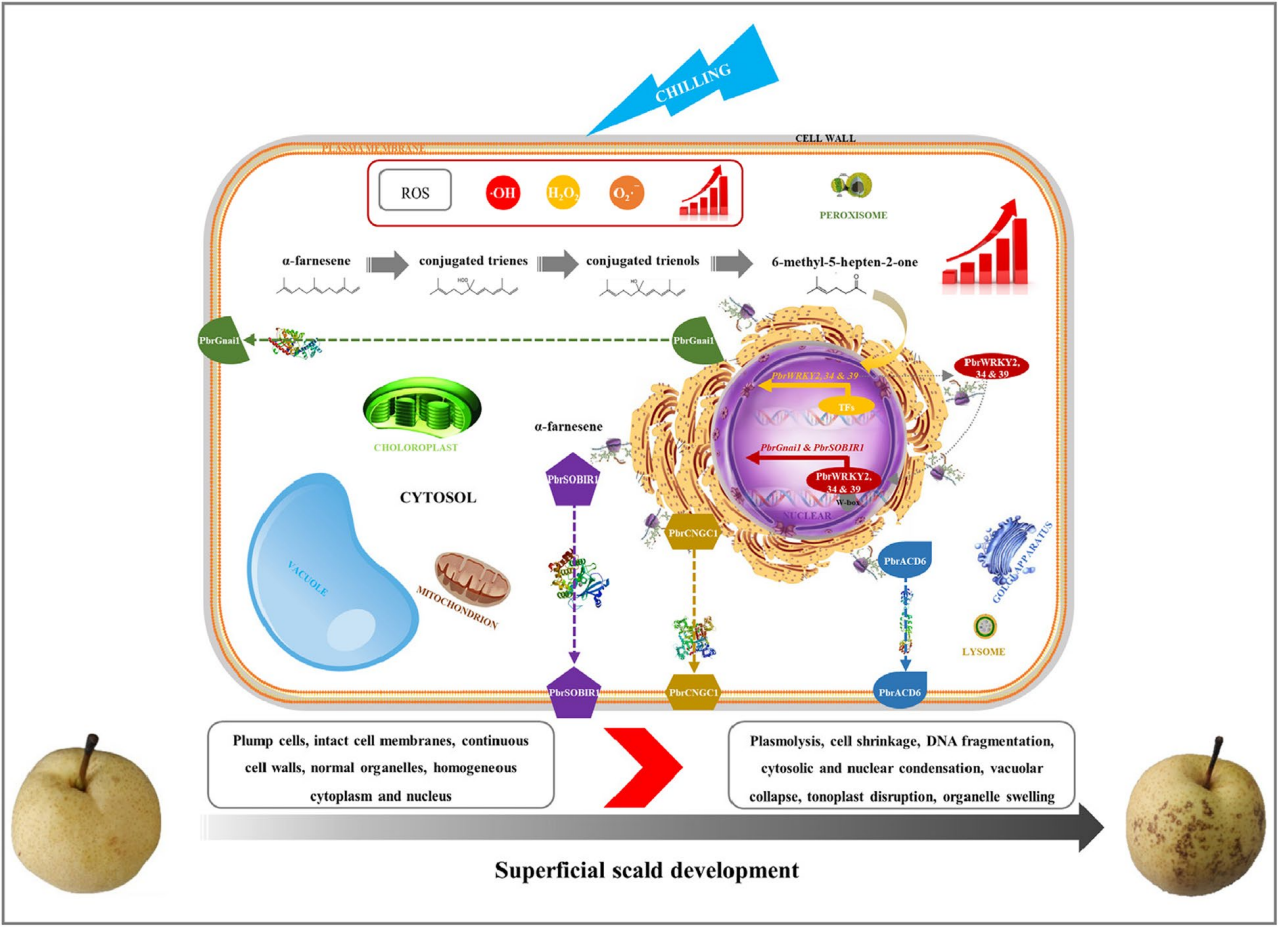
The schematic model of this study. Cold storage induces superficial scald development in pear fruit, which is accompanied by a bunch of typical PCD-associated morphological alterations, including plasmolysis, cell shrinkage, cytosolic and nuclear condensation, vacuolar collapse, tonoplast disruption, subcellular organelle swelling, and DNA fragmentation. These phenomena are associated with the alternation in the expression profiles of 24 PCD-related genes. MHO, as a signaling molecule, promotes PCD process, possibly through regulating the expression of *PbrGnai1*, *PbrSOBIR1*, *PbrACD6*, and *PbrCNGC1*; further study validated that three out of 23 TFs proposedly involved in MHO signaling pathway, including PbrWRKY2, 34, and 39, could interact with the W-box element in the promoter of *PbrGnai1* or *PbrSOBIR1* and then trigger their transcription

On the basis of the above, Fig. 7 presents the schematic model of this study. Cold storage induces superficial scald development in pear fruit, which is accompanied by a bunch of typical PCD-associated morphological alterations, including plasmolysis, cell shrinkage, cytosolic and nuclear condensation, vacuolar collapse, tonoplast disruption, subcellular organelle swelling, and DNA fragmentation. These phenomena are associated with the alternation in the expression profiles of 24 PCD-related genes. MHO, as a signaling molecule, promotes PCD process, possibly through regulating the expression of *PbrGnai1*, *PbrSOBIR1*, *PbrACD6*, and *PbrCNGC1*; further study validated that three out of 23 TFs proposedly involved in MHO signaling pathway, including PbrWRKY2, 34, and 39, could interact with the W-box element in the promoter of *PbrGnai1* or *PbrSOBIR1* and then trigger their transcription.

## Conclusion

The occurrence of scald symptom during cold storage of pear fruit was associated with a bunch of typical PCD-related morphological changes, whose formation was promoted by the signaling molecule MHO. Through transcriptome analysis, 24 PCD-related genes were characterized as the candidate members responsible for these cellular biological alternations upon scald development; among them, *PbrCNGC1*, *PbrGnai1*, *PbrACD6*, and *PbrSOBIR1* function through the MHO signaling pathway. Moreover, PbrWRKY2, 34, and 39 were validated to be the upstream regulators of *PbrGnai1* or *PbrSOBIR1*. Taken together, our study suggests that the activation of the PCD process, where MHO plays a positive role, is associated with scald development in pear fruit.

## Material and methods

### Materials and treatments

#### Experiment I

Uniform and defect-free *Pyrus bretschneideri* Rehd. cv. ‘Dangshansuli’ fruits, 1800 in total, were harvested from the homogeneous trees in a commercial orchard in Shaanxi Province, China. Their average weight, firmness, TSS, and TA were 226.58 g, 6.13 kg cm^-2^, 13.00 %, and 0.13 %, respectively. Upon transportation to the laboratory, fruits were randomly divided into three groups, with each treatment of 600 fruits (200 fruits per replicate × three biological replicates): (a) dipping in deionized distilled water for 60 s before storage at 0.5 ℃ (control); (b) dipping in 2.0 g L^-1^ DPA (Productos Citrosol S., Valencia, Spain; 31 %, w/v) for 60 s before storage at 0.5 ℃ (DPA dipping); (c) fumigation with 0.25 mL L^-1^ MHO for 48 h after 60-, 120-, and 180-d cold storage (MHO fumigation). Fruit samples were collected every 60 d, followed by 7-d shelf life at 20 ℃. For sampling, epidermal and sarcocarp tissues from eight fruits per replicate was quickly removed with a brass cork borer, mixed, and/or stored at -80 ℃ for quality assessment, physio-chemical measurement, and cellular biological investigation.

#### Experiment II

‘Dangshansuli’ fruits, uniform and defect-free, were harvested from homogeneous trees from an experimental orchard in Shaanxi Province, China. Fruits were immediately transported to the laboratory and then randomly divided into three treatments as described above. The epidermal tissues were collected every 60 d, followed by a 7-d shelf life at 25 ℃, for transcriptome and qRT-PCR assay.

#### Experiment III

‘Dangshansuli’ fruits, uniform and defect-free, were harvested from homogeneous trees from an experimental orchard in Shaanxi Province, China. Fruits were immediately transported to the laboratory and then randomly divided into four groups for the 48-h fumigation with 0.00 (control), 0.01, 0.25, and 1.00 mL L^-1^ MHO. The epidermal tissues were collected after a 7-d shelf life at 20 ℃ for qRT-PCR assay.

#### Experiment IV

‘Dangshansuli’ fruits, uniform and defect-free, were harvested from homogeneous trees from an experimental orchard in Shaanxi Province, China. Fruits were immediately transported to the laboratory and then randomly divided into five groups for 0.25 mL L^-1^ MHO fumigation for 0 (control), 4, 12, 24, and 48 h. The epidermal tissues were collected after a 7-d shelf life at 20 ℃ for qRT-PCR assay.

### Scald incidence and index assays

Scald incidence and index were determined based on a previous protocol (Hui et al. 2016). Scald grades were assigned as follows: Grade 0: no scald; Grade 1: scald area < 25 %; Grade 2: 25 % ≤ scald area < 50 %; Grade 3: scald area ≥ 50 %. Scald incidence and index were determined using the following formulas:

$$\text{Scald incidence }=\text{ Number of scalded fruit}/\text{Total number of fruit }\times 100\text{ \%}$$

$$\text{Scald index }= \sum [\text{Number of fruit }\times \text{ Grade}]/[\text{Total number of fruit }\times 3] \times 100\text{ \%}$$

### Firmness, TSS and TA assays

Firmness and TSS of the sarcocarp were measured by a fruit pressure tester (FT-327, Italy) and a digital refractometer (ATAGO PR-101, Atago Co., Tokyo, Japan), respectively (Li et al. 2021). Their results were expressed in unit of kg cm^-2^ and %, respectively.

For the determination of TA in sarcocarp tissue, sample was homogenized, filtered through two layers of Miracloth (Calbiochem, La Jolla, CA), and then centrifuged at 12, 000 *g* for 20 min to collect the supernatant for TA assay, using a titrator (808 Titrando, Metrohm, Riverview, FL, USA) (Li et al. 2021). The result was displayed in unit of %.

### ROS and α-farnesene-related metabolites assays

For H_2_O_2_ assay, the epidermal tissue was homogenized with 5.0 mL of 0.1 % trichloroacetic acid, and then centrifuged at 12, 000 *g* for 15 min at 4 ℃ to collect the supernatant. After the addition of 10 mmol L^-1^ pH 7.0 potassium phosphate buffer and 1 mol L^-1^ potassium iodide into the supernatant, H_2_O_2_ content was measured at 390 nm by a UV-Vis Spectrophotometer (UV-2450/2550, Shimadzu, Japan) (Velikova et al. 2000). The result was illustrated in unit of μmol g^-1^ FW.

O_2_‧^‾^ was analyzed following a previous method (Elstner and Heupel 1976). Briefly, the epidermal tissue was homogenized with 0.05 mol L^-1^ pH 7.8 phosphate buffer and 10 mmol L^-1^ hydroxylamine hydrochloride, and then centrifuged at 4, 000 *g* for 10 min at 4 ℃ to collect the supernatant. Afterwards, 17 mmol L^-1^ sulfanilamide and 7 mmol L^-1^ α-naphthylamine were added into the supernatant before O_2_‧^‾^ measurement using a UV-Vis Spectrophotometer (UV-2450/2550, Shimadzu, Japan) at 530 nm. The result was displayed in unit of μmol g^-1^ FW.

 ‧OH level assay was conducted following the method of Chomkitichai et al. (2014). Briefly, sixteen discs were incubated in the working solution containing 20 mmol L^-1^ pH 7.4 potassium phosphate and 2.8 mmol L^-1^ deoxyribose for 60 min. Subsequently, 1.0 mL of 0.5 % 2-thiobarbituric acid was added to the mixture, followed by incubation at 100 ℃ for 10 min. After cooling to room temperature, the fluorescence signals were measured using a microplate spectrophotometer (Synergy™ HT, BioTek Instruments, Inc., Winooski, VT, USA) with excitation at 530 nm and emission at 553 nm. The result was expressed in unit of nmol g^-1^ FW.

For the α-farnesene and conjugated trienes assays, the epidermis was homogenized with hexane. After transferring into a transparent glass-vial and then incubating at 23 ℃ for 20 min, the sample was centrifuged at 13, 000 *g* for 5 min before the analysis of α-farnesene content at 232 nm and conjugated trienes at 281-290 nm using a UV-Vis Spectrophotometer (UV-2450/2550, Shimadzu, Japan) (Ding et al. 2019). The results were demonstrated in unit of nmol g^-1^ FW.

MHO was determined using a Headspace, Solid-Phase Microextraction, and Gas Chromatographye-Mass Spectrometry (HS-SPME-GC-MS) method as described by Hui et al. (2016). Briefly, the epidermal tissue was homogenized with the saturated CaCl_2_ and then transferred to a 20-mL vial. After incubation at 40 ℃ for 30 min, MHO was assayed using a GC-MS system (Model 6890, Agilent, Santa Clara, CA, USA) equipped with DB-5 columns (60 m length, 0.25 mm i.d., 1.00 µm film thickness; J&W Scientific, Folsom, CA, USA) and a 5973 N MS detector (Agilent). Quantification of MHO was conducted by using a peak size vs. concentration curve using serially diluted five-point standard solutions, and the result was illustrated in unit of nmol μL kg^-1^ h^-1^ FW.

### Plasma membrane integrity and lipid peroxidation assays

Relative conductivity, an indicator of plasma membrane integrity, was measured as described previously (Feng et al. 2018). Briefly, sixteen discs were rinsed with redistilled water, and then transferred into a conical flask. After shaking for 20 min, electrolytic leakage was measured before and/or after a 100 ℃-water bath for 10 min using a conductivity meter (DDSJ-318T, INESA, China), and the result was expressed in unit of %.

Malondialdehyde (MDA), an indicator of plasma membrane lipid peroxidation, was quantified following the method of Feng et al. (2018). Briefly, epidermal tissue was homogenized with 5.0 mL of 0.1 % trichloroacetic acid before the addition of 0.5 % 2-thiobarbituric acid. After a 100 ℃-water bath for 10 min, the sample was centrifuged at 10, 000 *g* for 10 min at 4 ℃. Finally, the supernatant was collected for MDA measurement using a UV-Vis Spectrophotometer (UV-2450/2550, Shimadzu, Japan), and the result was displayed in unit of nmol g^-1^ FW.

### Cellular biological assays

#### Paraffin sectioning

About 1.0 cm^2^ epidermal tissue was fixed in FAA standard fixative for 24 h. Subsequently, the sample was washed with distilled water and sequentially immersed in 75-100 % ethanol and xylene-ethanol (v: v=1: 1). After waxing, the sample was transferred to a paper box for slicing.

#### Safranin O-fast green staining

Safranin O-fast green staining of paraffin-embedded tissue was performed using a previous protocol (Schuller and Ludwig-Müller 2016). After dewaxing, the sample was stained with 1 % safranin O solution, followed by an incubation in ethanol. Subsequently, the sample was stained with fast green solution for 10 s before the sequential incubation in xylene, xylene-ethanol, and ethanol. Finally, the sample was sealed with gum and then incubated at 40 ℃ for 1-2 d before microscopic analysis.

#### Toluidine blue O staining

Toluidine blue O staining of paraffin-embedded tissue was performed following the method of Schuller and Ludwig-Müller (2016). After dewaxing, the sample was stained with toluidine blue O stain and then incubated at 38 ℃ for 30 min. Subsequently, the sample was cleaned, dehydrated, blocked with neutral gum, and dried at 40 ℃ for 24 h before image capturing with a polarized light microscope (ZEISS, Germany).

#### TUNEL staining

TUNEL staining of paraffin-embedded tissue was carried out following a previous protocol (Vizcay-Barrena and Wilson 2006). After dewaxing and rehydration, the sample was immersed in Proteinase K, followed by a wash with phosphate buffered saline. Then, the sample was dropwise treated with a film-breaking solution and incubated at room temperature. Subsequently, a mixture of TUNEL enzyme (TdT) and TUNEL tag (dUTP) in a 1: 9 ratio was then applied to the sample, followed by a 37 °C incubation. Nuclear DNA was labelled with DAPI. Image capture was carried out using an ortho-fluorescence microscope (NIKON, Japan), with excitation at 330-380 nm for DAPI.

#### TEM analysis

TEM analysis of the epidermal tissue was performed following a previous method (Vizcay-Barrena and Wilson 2006). Briefly, the epidermal tissue was excised from the equatorial area and quickly fixed using electron microscope fixative (G1102, Servicebio, China). After a 2-h incubation at room temperature, the tissue was rinsed with 0.1 mol L^-1^ PBS (pH 7.4) and post-fixed with 1 % OsO_4_. Then, the sample was sequentially dehydrated with a graded ethanol series for resin penetration, embedding, polymerization and ultrathin section. Finally, the sample was stained with 2 % uranyl acetate and 2.6 % lead citrate before image capture (Hitachi, HT7800/HT7700, Japan).

### Transcriptome and qRT-PCR analysis

Transcriptome analysis was conducted following the previous method (Li et al. 2019a and 2019b). Briefly, total RNA was extracted from the epidermal tissue, followed by RNA concentration and integrity assays. Subsequently, 5.0 μg RNA was used for constructing the complementary DNA (cDNA) library and then sequenced on the BGISEQ-500 platform (BGI, Shenzhen, China). After removing adapter sequences and low-quality reads, the clean reads were aligned to the *P. bretschneideri* genome (Wu et al. 2013; Li et al. 2019a and 2019b). Gene expression was quantified using FPKM, and differentially expressed genes (DEGs) were identified using the NOISeq software, applying the following criteria: fold change ≥ 1.5 and FDR < 0.05 (Li et al. 2019a and 2019b).

qRT-PCR analysis was conducted based on the protocol of Wang et al. (2018), using gene-specific primers (Table S1). Briefly, total RNA was isolated, followed by RNA integrity, concentration, and purity assays. Then, first-strand cDNA synthesis was performed using the TransScript^®^ One-Step gDNA Removal and cDNA Synthesis SuperMix (TRANSGEN, China). qRT-PCR assays were performed using the SYBR® PrimeScript™ RT-PCR Kits (Perfect Real Time, Takara). *Pyrus* tubulin genes were used as the housekeeping genes, and the relative gene expression was calculated using the 2^-ΔΔCt^ method.

### Bioinformation analysis

The physio-biochemical parameters of proteins were calculated using the ProtParam tool (https://web.expasy.org/protparam/) (Zhang et al. 2021). Subcellular localizations were predicted by the ProtComp v. 9.0 database (http://linux1.softberry.com/berry.phtml?topic=protcomppl&group=programs&subgroup=proloc) (Zhao et al. 2018). Transmembrane helices were analyzed using the TMHMM-2.0 Server (https://services.healthtech.dtu.dk/service.php?TMHMM-2.0) (Zhao et al. 2018). Signal peptides were analyzed with the aid of the SignalP 5.0 Server (https://services.healthtech.dtu.dk/service.php?SignalP-5.0) (Ma et al. 2020). *Cis*-acting elements in gene promoters were identified by the PlantCARE database (http://bioinformatics.psb.ugent.be/webtools/plantcare/html/) (Zhang et al. 2021). On the other hand, the binding site of TF in the promoter was predicted via the PlantRegMap database (http://plantregmap.gao-lab.org/) (Tian et al. 2020).

### Subcellular localization assay

The coding sequence of *PbrCNGC1* and *PbrSOBIR1*, without stop codons, were cloned from ‘Dangshansuli’ fruits (Table S1), and inserted into the pBI221 vector containing a GFP tag. The constructs were then co-transformed with plasma membrane marker OsMCA1-mcherry into the protoplasts of *O. sativa* etiolated seedling (Kurusu et al. 2012; Ma et al. 2020). Extraction of protoplast from *O. sativa* etiolated seedling was conducted according to a previous method (Ma et al. 2020).

To determine the subcellular localization of PbrWRKY2, 34, and 39, their coding sequences were amplified into the pBI221 vector possessing a GFP tag (Table S1). The constructs were then injected into *N. benthamiana* leaves following the protocol of Lin et al. (2022). DAPI was used as a nuclear indicator (Kapuscinski 1995). Fluorescence signal was detected using a confocal microscope (Leica Microsystems, Germany).

### Dual-LUC assay

The coding sequences of *PbrWRKY2*, *34* and *39* were amplified from ‘Dangshansuli’ fruit, and then introduced into the pCAMBIA1300 vector (Table S1). On the other hand, the promoters of *PbrCNGC1*, *PbrGnai1*, and *PbrSOBIR1*, containing the wide-type W-box elements (core motif: TTGACC/T; *PbrCNGC1pro*, *PbrGnai1pro*, and *PbrSOBIR1pro*) or the mutated elements (TTGACC/T→TTTAGC/T, *PbrCNGC1pro*^*mut*^, *PbrGnai1pro*^*mut*^, and *PbrSOBIR1pro*^*mut*^), were inserted into the pGreenII 0800-LUC vector (Table S1), producing various reporters. Subsequently, a mixture of *A. tumefaciens* containing pCAMBIA1300-*PbrWRKY2/34/39* vector and each reporter was infiltrated into *N. benthamiana* leaves. Co-transformants containing pCAMBIA1300 & pGreen 0800-LUC vectors, pCAMBIA1300 & pGreen 0800-*PbrCNGC1pro*/*PbrGnai1pro*/*PbrSOBIR1pro/PbrCNGC1pro*^*mut*^/*PbrGnai1pro*^*mut*^/*PbrSOBIR1pro*^*mut*^-LUC vectors, or pCAMBIA1300-*PbrWRKY2/34/39* & pGreen 0800-LUC vectors were used as control. LUC image was captured by a Chemiluminescence Imager (SH-Compact523, SHST, China); on the other hand, LUC and Renilla (REN) activities were determined using a dual-LUC reporter assay system (Promega, Madison, WI, USA) (Liu et al. 2022b).

### Y1H assay

The coding sequences of *PbrWRKY2*, *34* and *39* were amplified and cloned into the prey vector pGADT7. Additionally, a 200-bp fragment of *PbrGnai1* and *PbrSOBIR1* promoter, containing the wide-type W-box elements (core motif: TTGACC/T; *PbrGnai1pro* and *PbrSOBIR1pro*) or the mutated elements (TTGACC/T→TTTAGC/T, *PbrGnai1pro*^*mut*^ and *PbrSOBIR1pro*^*mut*^), was inserted into bait vector pAbAi (Table S1). Y1H assay was performed using Matchmaker Gold Yeast One-Hybrid Library Screening System (Weidi, Shanghai, China) (Jian et al. 2019). D/-Ura medium supplemented with Aureobasidin A (AbA) was used to examine *PbrGnai1pro*, *PbrSOBIR1pro*, *PbrGnai1pro*^*mut*^, and *PbrSOBIR1pro*^*mut*^ self-activation and to select the proper AbA concentration. Yeast cell co-transformed with AD-*p53* & *p53*-AbAi was used as the positive control, while yeasts co-transformed with the empty AD vector and each bait as the negative controls.

### Gene function validation *in vivo*

#### Transient overexpression of genes in pear epidermis

The coding sequences of *PbrWRKY2*, *34*, and *39* genes after amplification from ‘Dangshansuli’ fruit were introduced into the pCAMBIA1300 vector (Table S1), transformed into *A. tumefaciens* strain GV3101, and then incubated at 28 °C until OD_660_ reached 1.0. After centrifugation and resuspension of the bacterial strain in an infiltration buffer (10 mmol L^-1^ MgCl_2_, 10 mmol L^-1^ MES (pH 5.5) and 150 μmol L^-1^ acetosyringone), 10 μL of solution was slowly injected into the epidermis of ‘Dangshansuli’ fruits. Epidermal tissue from the injection sites was collected after 3-d storage at 25 °C. Fruits infiltrated with empty pCAMBIA1300 vector were used as controls (Ma et al. 2020). There were three biological replicates per treatment, with eight fruit per biological replicate.

#### Transient silence of genes in pear epidermis

About 250-bp fragments of *PbrWRKY2*, *34*, and *39* genes were amplified and then inserted into the pTRV2 vector (Table S1). The constructed plasmids, along with pTRV1, were separately transformed into *Agrobacterium tumefaciens* strain GV3101. Afterward, the bacterial resuspensions containing recombinant pTRV2 and pTRV1 were mixed in a 1:1 ratio before injection into the epidermal tissue of the ripe ‘Dangshansuli’ fruit. The epidermal tissue from the injection sites was collected after 3-d storage in the dark at 25 °C. The empty pTRV2 vector, co-injected with pTRV1, was used as a control (Zhang et al. 2019). There were three biological replicates per treatment, with eight fruit per biological replicate.

### Statistical analysis

The data represented the mean values of three biological replicates and one-way analysis of variance (ANOVA) was conducted at a significance of *P* < 0.05. Data analysis was performed using SAS version 9.3 (SAS Institute, Cary, NC). Graphs were generated using GraphPad Prism 8.0.2 and RStudio software. R package was used to calculate the Pearson correlation coefficient between attributes, where the extremely strong correlation was in the range of 0.8-1.0, and the strong correlation was in the range of 0.6-0.8 (Long et al. 2014).

### Supplementary Information

Supplementary Material 1: Table S1. Primers used in this study. Table S2. Expression profiles (FPKMs) of 146 PCD-related genes during cold storage of pear fruit based on the transcriptome result of Experiment II. ‘Dangshansuli’ fruits were randomly divided into three treatments: H_2_O dipping (control), MHO fumigation, and DPA dipping. The samples were collected every 60 d followed by a 7-d shelf life at 25 ℃. 146 PCD-related genes were identified based on transcriptome annotation. Data, adapted from transcriptome assay, present the mean value of three biological replicates. Table S3. Information of PbrCNGC1, PbrGnai1, PbrACD6, and PbrSOBIR1. Physio-biochemical parameters of proteins were calculated by the ProtParam tool (Zhang et al. 2021). Subcellular localizationswere predicted by the ProtComp v. 9.0 database (Zhao et al. 2018). The conserved domains were identified by the SMART database (Zhang et al. 2021). Table S4. Information on W-boxes, G-boxes, and MYB-binding sites in *PbrCNGC1*, *PbrGnai1*, *PbrACD6*, and *PbrSOBIR1*promoters. W-boxes, G-boxes, and MYB-binding sites in *PbrCNGC1*, *PbrGnai1*, and *PbrACD6 *promoters were predicted by the PlantCARE database (Zhang et al. 2021). Table S5. Expression profiles (FPKMs) of TFs during cold storage of pear fruit. ‘Dangshansuli’ fruits were randomly divided into three treatments: H_2_O dipping (control), MHO fumigation, and DPA dipping. The samples were collected every 60 d followed by a 7-d shelf life at 25 ℃. Data adapted from transcriptome assay, present the mean value of three biological replicates. Table S6. Possible binding sites of TFs in the promoters of* PbrCNGC1*, *PbrGnai1*, *PbrACD6*, and *PbrSOBIR1*. The homologous proteins of PbrWRKYs, PbrbZIPs, and PbrMYBs from *Arabidopsis *were identified with the aid of the Plant Transcription Factor Database (http://planttfdb.gao-lab.org/prediction.php). Afterwards, the possible binding sites of the homologous proteins from *Arabidopsis* in the promoters of* PbrCNGC1*, *PbrGnai1*, and *PbrACD6 *were characterized by the JASPAR CORE database (https://jaspar.genereg.net/).

Supplementary Material 2: Fig. S1. Dynamic changes of superficial scald during cold storage of pear fruits. (a) Scald incidence; (b) scald index. ‘Dangshansuli’ fruits were randomly divided into three treatments: H_2_O dipping (control), MHO fumigation, and DPA dipping. The samples were collected every 60 d followed by a 7-d shelf life at 20 ℃. The data are the mean values ± SD of three biological replicates. Vertical bars labeled with the same letter indicate no significant difference between samples at the same sampling time at *P < *0.05. Fig. S2. Dynamic change of firmness, TSS, and TA in sarcocarp tissue during cold storage of pear fruit. (a) Firmness; (b) total soluble solids; (c) titratable acids. ‘Dangshansuli’ fruits were randomly divided into three treatments: H_2_O dipping (control), MHO fumigation, and DPA dipping. The samples were collected every 60 d followed by a 7-d shelf life at 20 ℃. The data are the mean values ± SD of three biological replicates. Vertical bars labeled with the same letter indicate no significant difference between samples at the same sampling time at *P< *0.05. Fig. S3. Correlations among attributes. ‘Dangshansuli’ fruits were randomly divided into three treatments: H_2_O dipping (control), MHO fumigation, and DPA dipping. The samples were collected every 60 d followed by a 7-d shelf life at 20 ℃. Pearson correlations among attributes are visualized as a heatmap;^*^ and ^**^ represent significance at *P* < 0.05 and 0.01, respectively. Fig. S4. TEM analysis of pear fruits. ‘Dangshansuli’ fruits were randomly divided into three treatments: H_2_O dipping (control), MHO fumigation, and DPA dipping. The samples were collected every 60 d followed by a 7-d shelf life at 20 ℃. Abbreviations: Chl, chloroplast; CW, cell wall; M, mitochondria; N, nucleus; RER, endoplasmic reticulum; T, tonoplast; V, vacuole; ▲, plasmolysis. Fig. S5. Alignment of plant CNGC1s, Gnai1s, ACD6s, and SOBIR1s. Protein information on CNGC1s, Gnai1s, ACD6s, and SOBIR1s from other plants (*O. sativa* (Os), *Arabidopsis* (At), and *N. tabacum* (Nt)) were reported in the previous reports (Li et al. 2017; Wang et al. 2018; Giampieri et al. 2018). Alignment of protein sequences was conducted with the aid of the DNAman software. Fig. S6. Bioinformation analysis of PbrCNGC1, PbrGnai1, PbrACD6, and PbrSOBIR1. Transmembrane helices were analyzed by the TMHMM-2.0 Server (Wang et al., 2018). Signal peptides were assayed by the SignalP 5.0 Server (Ma et al. 2020). Protein 3D-structures were predicted by the SWISS-MODEL Server (Wang et al. 2018). Fig. S7. Dual-LUC assay for the inactivation of* PbrSOBIR1* and *PbrCNGC1* expression by PbrWRKY2, 34 and 39. Co-transformants containing pCAMBIA1300 & pGreen 0800-LUC vectors, pCAMBIA1300& pGreen 0800-gene promoter-LUC vectors, or pCAMBIA1300-TF & pGreen 0800-LUC vectors were used as control. The data are the mean values ± SD of three biological replicates. Vertical bars labeled with the same letter indicate no significant difference between samples at the same sampling time at*P < *0.05. Fig. S8. Impact of mutation of W-box elements in *PbrGnai1* and *PbrSOBIR1* promoters on their interaction with (or activation by) *PbrWRKY2*,* 34*, and* 39*. (a) Dual-LUC assay. Co-transformants containing pCAMBIA1300 & pGreen 0800-LUC vectors, pCAMBIA1300 & pGreen 0800-*PbrGnai1pro*/*PbrSOBIR1pro/PbrGnai1pro*^*mut*^/*PbrSOBIR1pro*^*mut*^-LUC vectors, or pCAMBIA1300-*PbrWRKY2/34/39* & pGreen 0800-LUC vectors were used as control. The data are the mean values ± SD of three biological replicates, and vertical bars labelled labeled with the same letter indicate no significant difference between samples at *P < *0.05. (b) Y1H assay. Yeast cell co-transformed with AD-*p53* & *p53*-AbAi was used as the positive control, while yeasts co-transformed with the empty AD vector and each bait as the negative controls. Fig. S9. Impact of transient transformation of pear fruit on *PbrWRKY2*, *34* and *39* expression abundances. Fruit transformed with the empty pCAMBIA1300 vector was used as a control for TF-overexpressing fruit, while fruit co-transformed with empty TRV2 and TRV1 was used as a control for TF-silenced fruit. Expression abundance of each gene in control fruit was set as 1.0 based on qRT-PCR result. The data are the mean values ± SD of three biological replicates. Vertical bars labeled with the same letter indicate no significant difference between samples at the same sampling time at*P < *0.05. Fig. S10. Impact of MHO fumigation concentration on scald development and *PbrWRKY2*, *34* and *39* expression levels in ‘Dangshansuli’ fruit. (a) Visual quality. (b) Scald incidence and index. (c) *PbrWRKY2*, *34* and *39* expression levels. (d) Correlations among attributes. ‘Dangshansuli’ fruits were randomly divided into four groups for the 48-h fumigation with 0.00 (control), 0.01, 0.25, and 1.00 mL L^-1^ MHO, prior to a 7-d shelf life at 20 ℃. Expression abundance of each gene in control fruit was set as 1.0 based on qRT-PCR result. The data are the mean values ± SD of three biological replicates. Vertical bars labeled with the same letter indicate no significant difference between samples at the same sampling time at *P<* 0.05. Fig. S11. Impact of MHO fumigation time on scald development and *PbrWRKY2*, *34* and *39* expression levels in ‘Dangshansuli’ fruit. (a) Visual quality. (b) Scald incidence and index. (c) *PbrWRKY2*, *34* and *39* expression levels. (d) Correlations among attributes. ‘Dangshansuli’ fruits were randomly divided into five groups for 0.25 mL L^-1^MHO fumigation for 0 (control), 4, 12, 24, and 48 h, prior to a 7-d shelf life at 20 ℃. Expression abundance of each gene in control fruit was set as 1.0 based on qRT-PCR result. The data are the mean values ± SD of three biological replicates. Vertical bars labeled with the same letter indicate no significant difference between samples at the same sampling time at*P < *0.05.

## Data Availability

The authors confirm that all data in this study are included in this published article (and its Supplementary information file).

## Abbreviations

1-MCP: 1-methylcyclopropene
AbA: Aureobasidin A
ATP: Adenosine triphosphate
Cyt c: Cytochrome c
DAD1: Defender against cell death 1
DAPI: 4’, 6-diamidino-2-phenylindole
DND1: Defense, no death 1
DEGs: Differentially expressed genes
DPA: Diphenylamine
FDR: False discovery rate
FPKM: Fragments per kilobase million
H_2_O_2_: Hydrogen peroxide
LSD1: Lesion simulating disease 1
LUC: Luciferase
MHO: 6-methyl-5-hepten-2-one
NO: Nitric oxide
O_2_·^¯^: Superoxide anion
‧OH: Hydroxyl free radical
PbrACD6: Accelerated cell death 6-like protein
PbrCNGC1: Cyclic nucleotide-gated ion channel 1-like protein
PbrGnai1: Guanine nucleotide-binding protein alpha-1 subunit
PbrSOBIR1: Leucine-rich repeat receptor-like serine/threonine/tyrosine-protein kinase sobir1
PCD: Programmed cell death
qRT-PCR: Quantitative real-time polymerase chain reaction
REN: Renilla
ROS: Reactive oxygen species
TA: Titratable acids
TEM: Transmission electron microscope
TFs: Transcription factors
TSS: Total soluble solid
TUNEL: TdT-mediated dUTP nick-end labeling
Y1H: Yeast one-hybrid
