# Supplementary material for: 6-Methyl-5-hepten-2-one promotes programmed cell death during superficial scald development in pear
Source: Mol Hortic. 2024 Aug 27;4:32. doi: 10.1186/s43897-024-00107-1 (PMC11348602; doi:10.1186/s43897-024-00107-1)
Supplement: Supplementary file 2 — Supplementary Material 2: Fig. S1. Dynamic changes of superficial scald during cold storage of pear fruits. (a) Scald incidence; (b) scald index. ‘Dangshansuli’ fruits were randomly divided into three treatments: H2O dipping (control), MHO fumigation, and DPA dipping. The samples were collected every 60 d followed by a 7-d shelf life at 20 ℃. The data are the mean values ± SD of three biological replicates. Vertical bars labeled with the same letter indicate no significant difference between samples at the same sampling time at P < 0.05. Fig. S2. Dynamic change of firmness, TSS, and TA in sarcocarp tissue during cold storage of pear fruit. (a) Firmness; (b) total soluble solids; (c) titratable acids. ‘Dangshansuli’ fruits were randomly divided into three treatments: H2O dipping (control), MHO fumigation, and DPA dipping. The samples were collected every 60 d followed by a 7-d shelf life at 20 ℃. The data are the mean values ± SD of three biological replicates. Vertical bars labeled with the same letter indicate no significant difference between samples at the same sampling time at P< 0.05. Fig. S3. Correlations among attributes. ‘Dangshansuli’ fruits were randomly divided into three treatments: H2O dipping (control), MHO fumigation, and DPA dipping. The samples were collected every 60 d followed by a 7-d shelf life at 20 ℃. Pearson correlations among attributes are visualized as a heatmap;* and ** represent significance at P < 0.05 and 0.01, respectively. Fig. S4. TEM analysis of pear fruits. ‘Dangshansuli’ fruits were randomly divided into three treatments: H2O dipping (control), MHO fumigation, and DPA dipping. The samples were collected every 60 d followed by a 7-d shelf life at 20 ℃. Abbreviations: Chl, chloroplast; CW, cell wall; M, mitochondria; N, nucleus; RER, endoplasmic reticulum; T, tonoplast; V, vacuole; ▲, plasmolysis. Fig. S5. Alignment of plant CNGC1s, Gnai1s, ACD6s, and SOBIR1s. Protein information on CNGC1s, Gnai1s, ACD6s, and SOBIR1s from other pla [file 43897_2024_107_MOESM2_ESM.docx]

**Supplementary information**


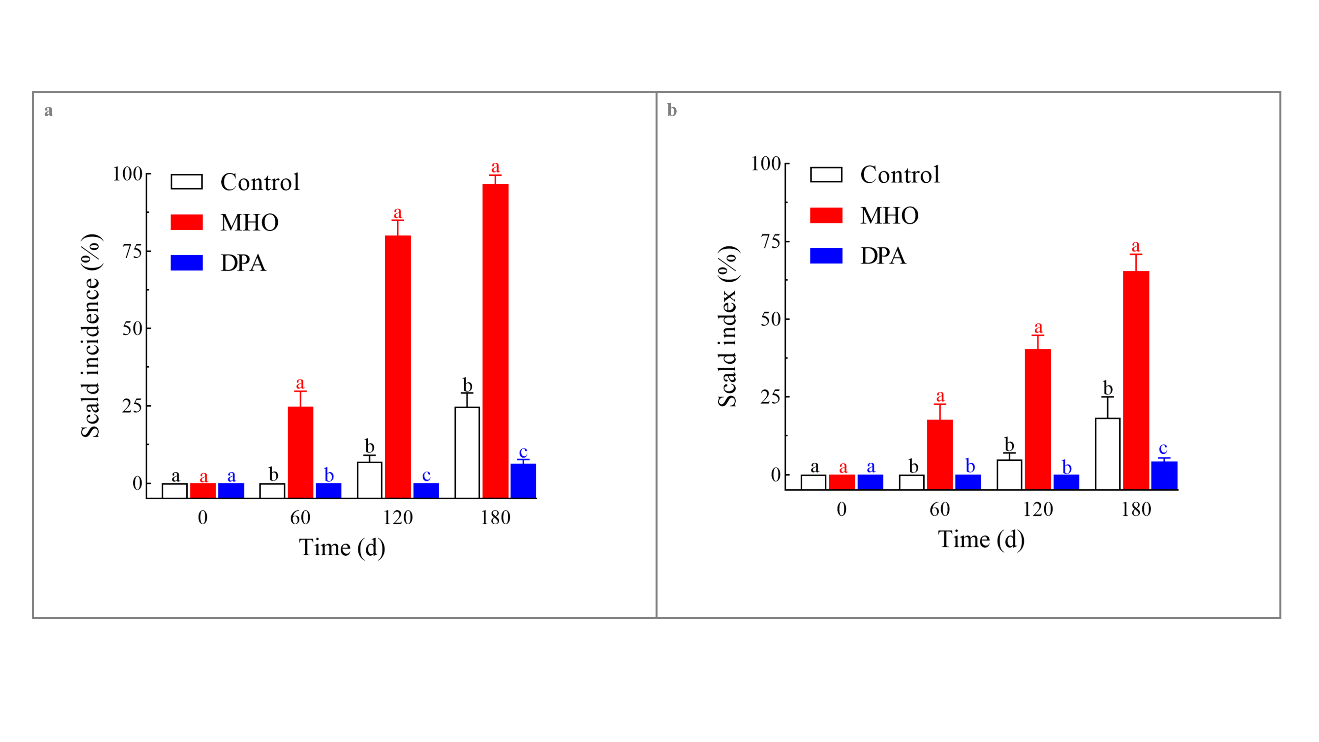


**Fig. S1 Dynamic changes of superficial scald during cold storage of pear fruits. (a) Scald incidence; (b) scald index.** ‘Dangshansuli’ fruits were randomly divided into three treatments: H_2_O dipping (control), MHO fumigation, and DPA dipping. The samples were collected every 60 d followed by a 7-d shelf life at 20 ℃. The data are the mean values ± SD of three biological replicates. Vertical bars labeled with the same letter indicate no significant difference between samples at the same sampling time at *P <* 0.05


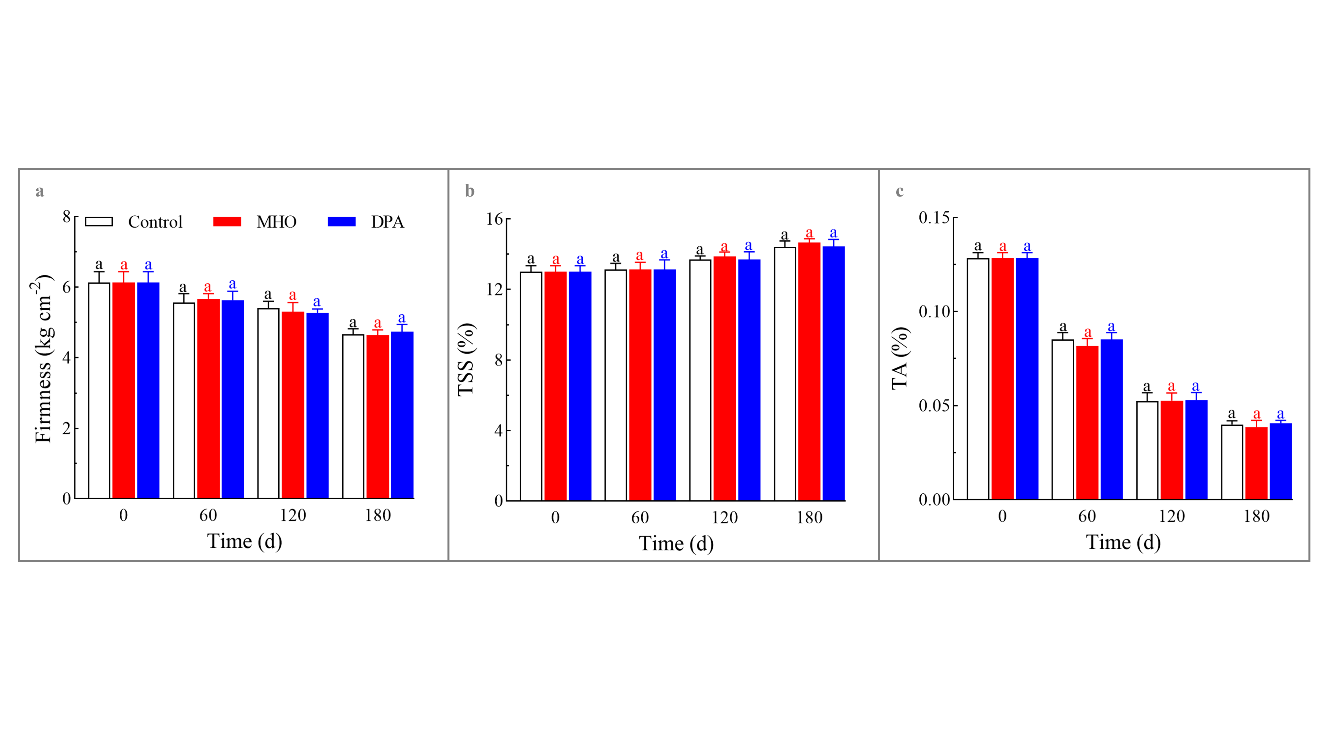


**Fig. S2 Dynamic change of firmness, TSS, and TA in sarcocarp tissue during cold storage of pear fruit. (a) Firmness; (b) total soluble solids; (c) titratable acids.** ‘Dangshansuli’ fruits were randomly divided into three treatments: H_2_O dipping (control), MHO fumigation, and DPA dipping. The samples were collected every 60 d followed by a 7-d shelf life at 20 ℃. The data are the mean values ± SD of three biological replicates. Vertical bars labeled with the same letter indicate no significant difference between samples at the same sampling time at *P <* 0.05


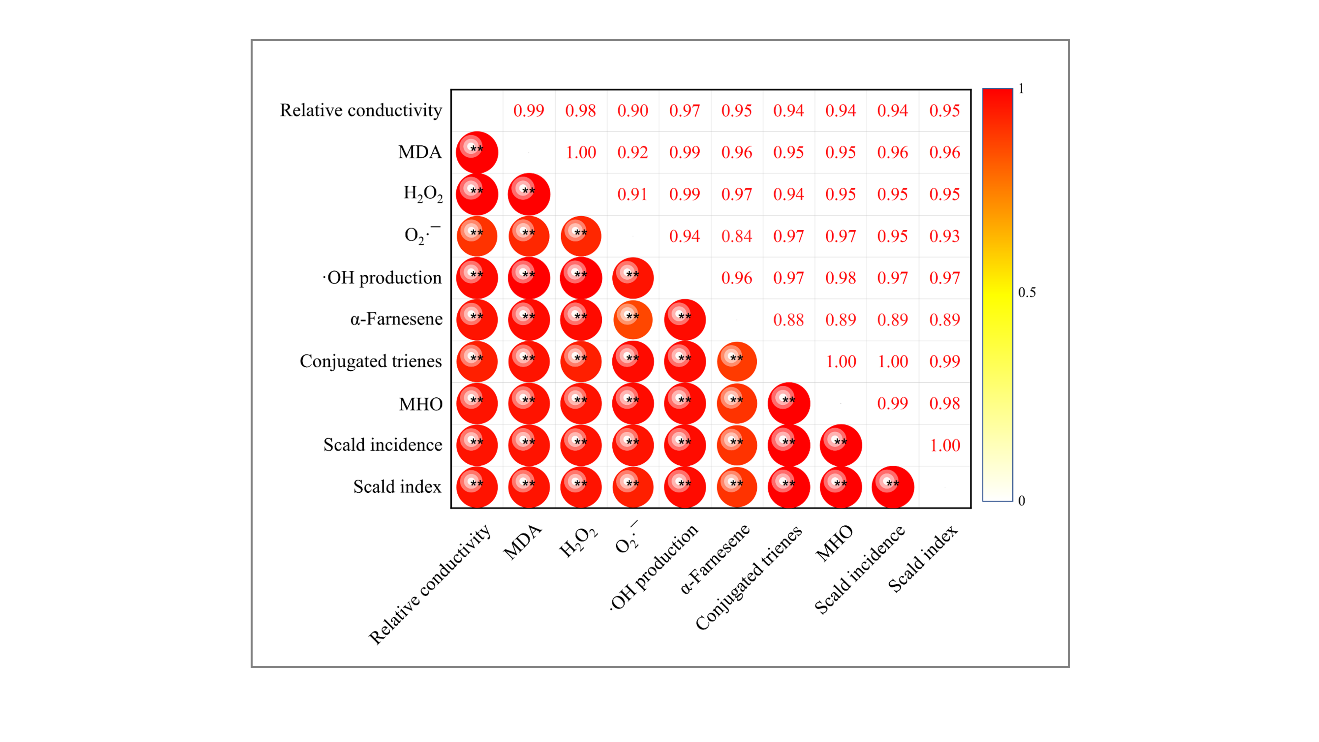


**Fig. S3 Correlations among attributes.** ‘Dangshansuli’ fruits were randomly divided into three treatments: H_2_O dipping (control), MHO fumigation, and DPA dipping. The samples were collected every 60 d followed by a 7-d shelf life at 20 ℃. Pearson correlations among attributes are visualized as a heatmap; ^*^ and ^**^ represent significance at *P* < 0.05 and 0.01, respectively


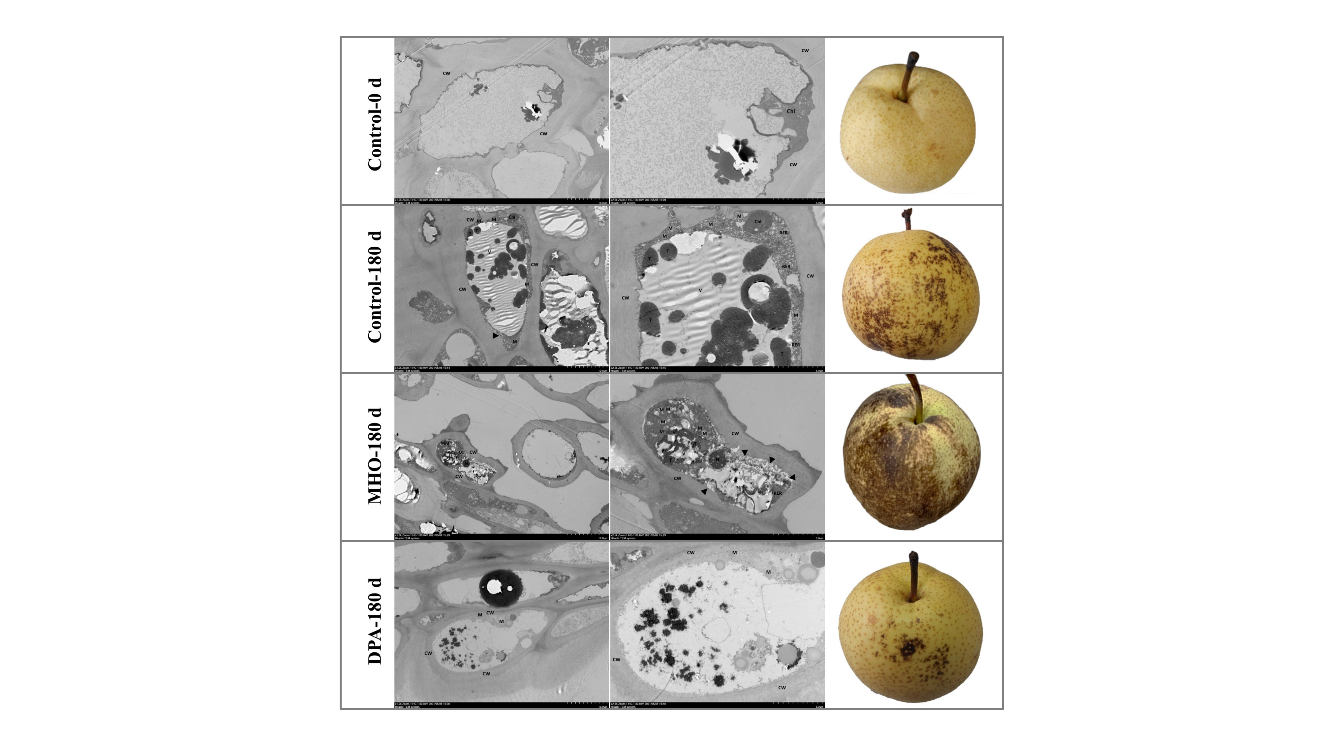


**Fig. S4 TEM analysis of pear fruits.** ‘Dangshansuli’ fruits were randomly divided into three treatments: H_2_O dipping (control), MHO fumigation, and DPA dipping. The samples were collected every 60 d followed by a 7-d shelf life at 20 ℃. Abbreviations: Chl, chloroplast; CW, cell wall; M, mitochondria; N, nucleus; RER, endoplasmic reticulum; T, tonoplast; V, vacuole; ▲, plasmolysis


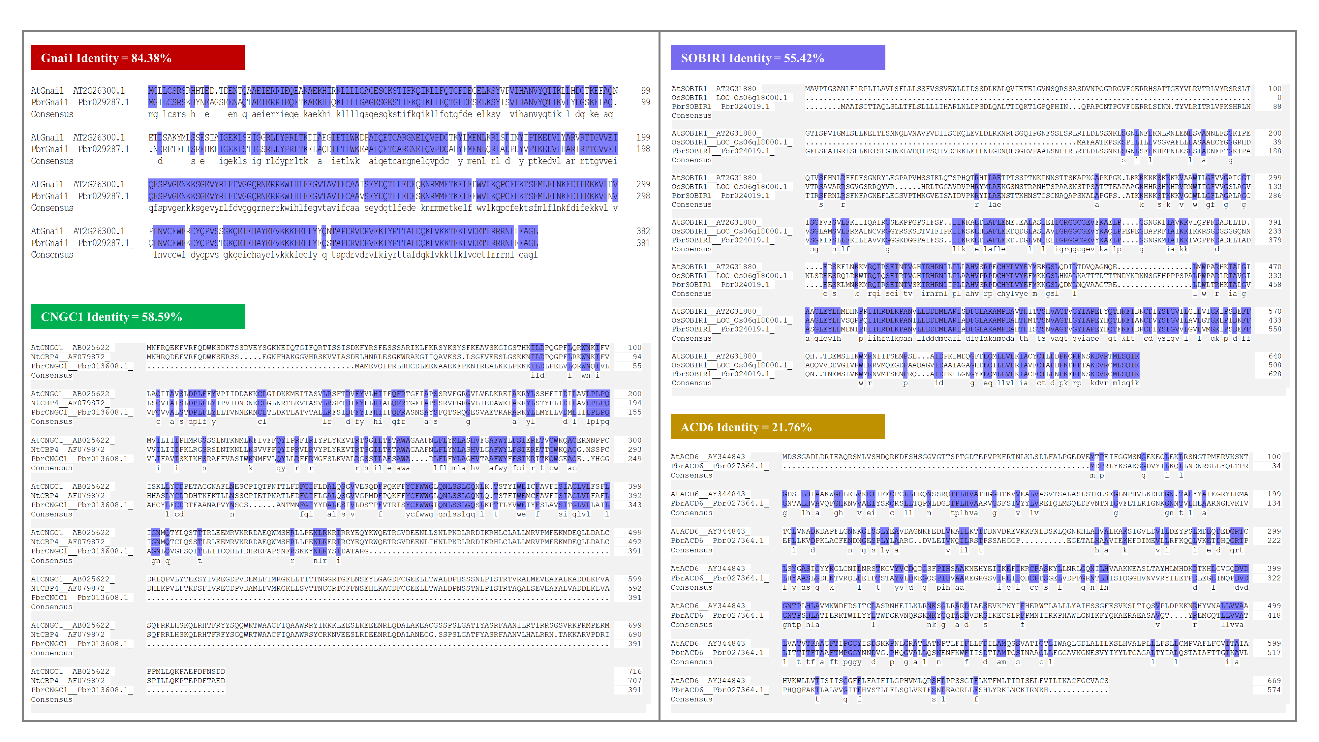


**Fig. S5 Alignment of plant CNGC1s, Gnai1s**, **ACD6s, and SOBIR1s.** Protein information on CNGC1s, Gnai1s, ACD6s, and SOBIR1s from other plants (*O. sativa* (Os), *Arabidopsis* (At), and *N. tabacum* (Nt)) were reported in the previous reports (Li et al. 2017; Wang et al. 2018; Giampieri et al. 2018). Alignment of protein sequences was conducted with the aid of the DNAman software


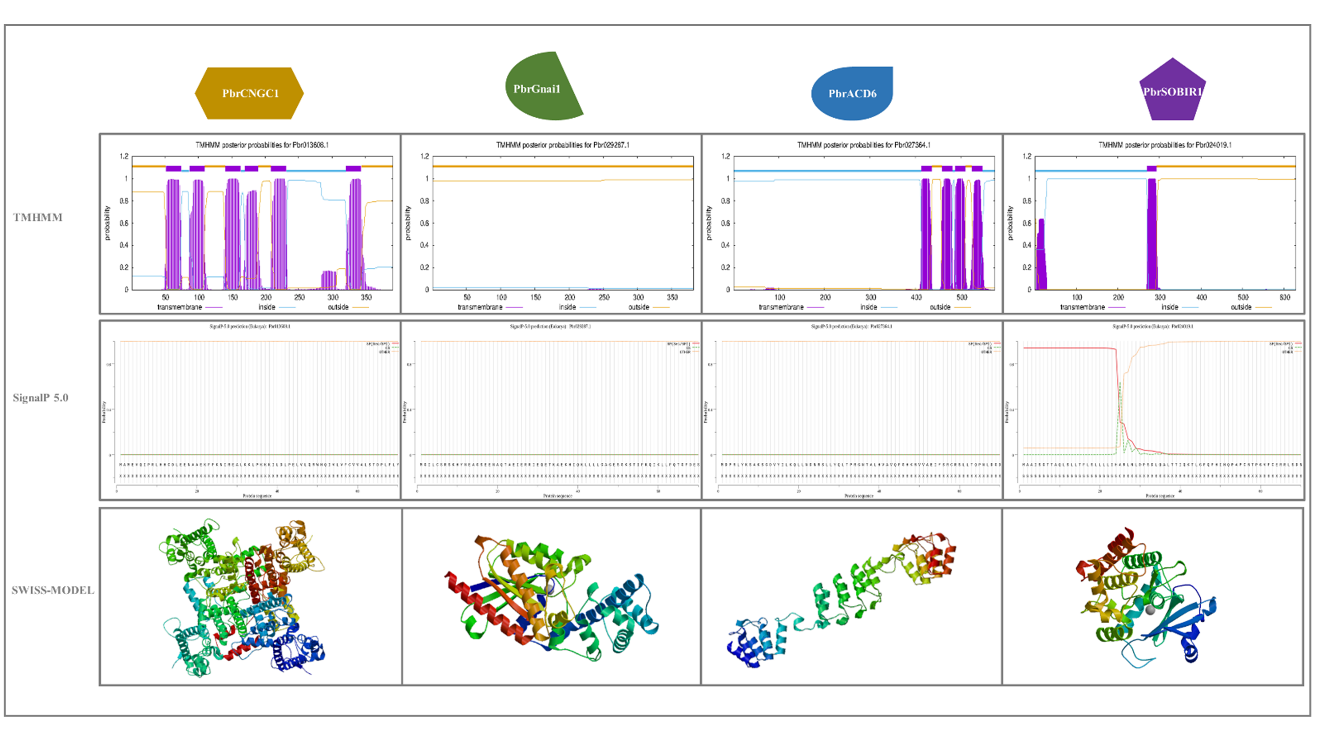


**Fig. S6 Bioinformation analysis of PbrCNGC1, PbrGnai1**, **PbrACD6, and PbrSOBIR1.** Transmembrane helices were analyzed by the TMHMM-2.0 Server (Wang et al., 2018). Signal peptides were assayed by the SignalP 5.0 Server (Ma et al. 2020). Protein 3D-structures were predicted by the SWISS-MODEL Server (Wang et al. 2018)


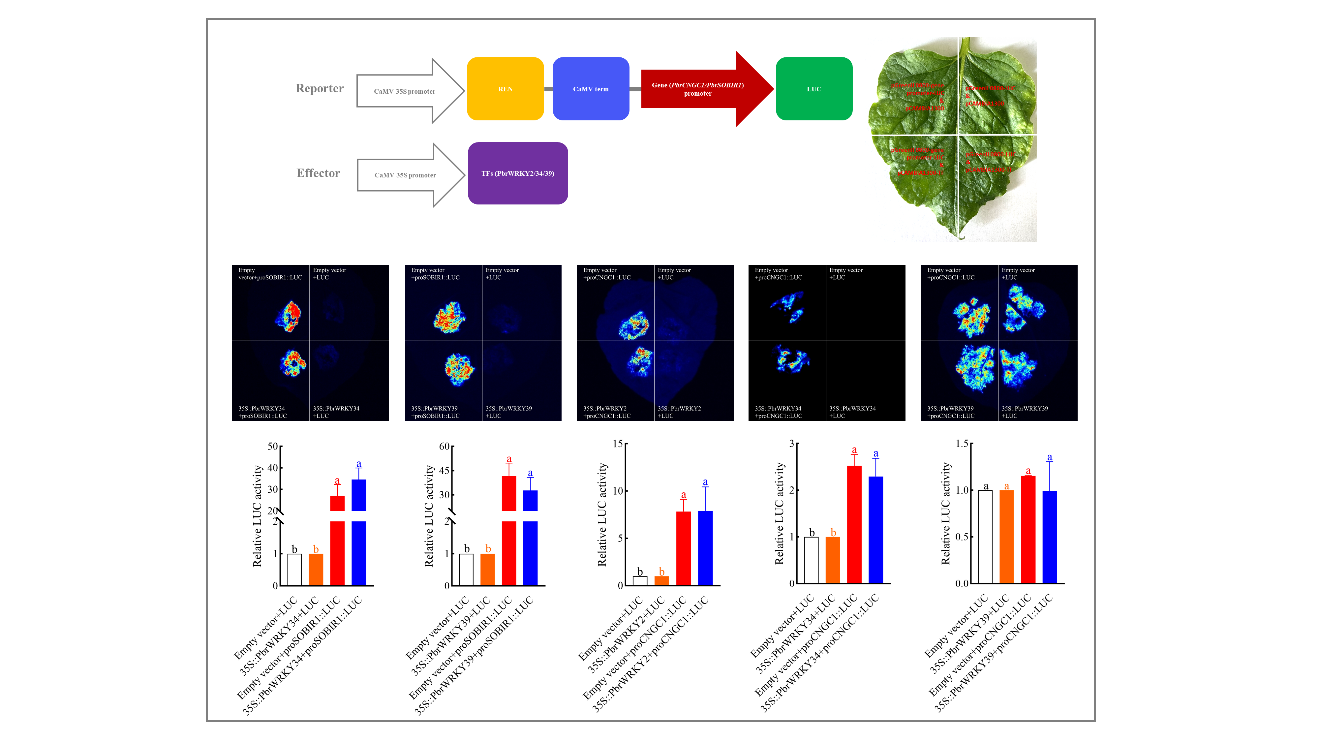


**Fig. S7 Dual-LUC assay for the inactivation of *PbrSOBIR1* and *PbrCNGC1* expression by PbrWRKY2, 34 and 39.** Co-transformants containing pCAMBIA1300 & pGreen 0800-LUC vectors, pCAMBIA1300 & pGreen 0800-gene promoter-LUC vectors, or pCAMBIA1300-TF & pGreen 0800-LUC vectors were used as control. The data are the mean values ± SD of three biological replicates. Vertical bars labeled with the same letter indicate no significant difference between samples at the same sampling time at *P <* 0.05


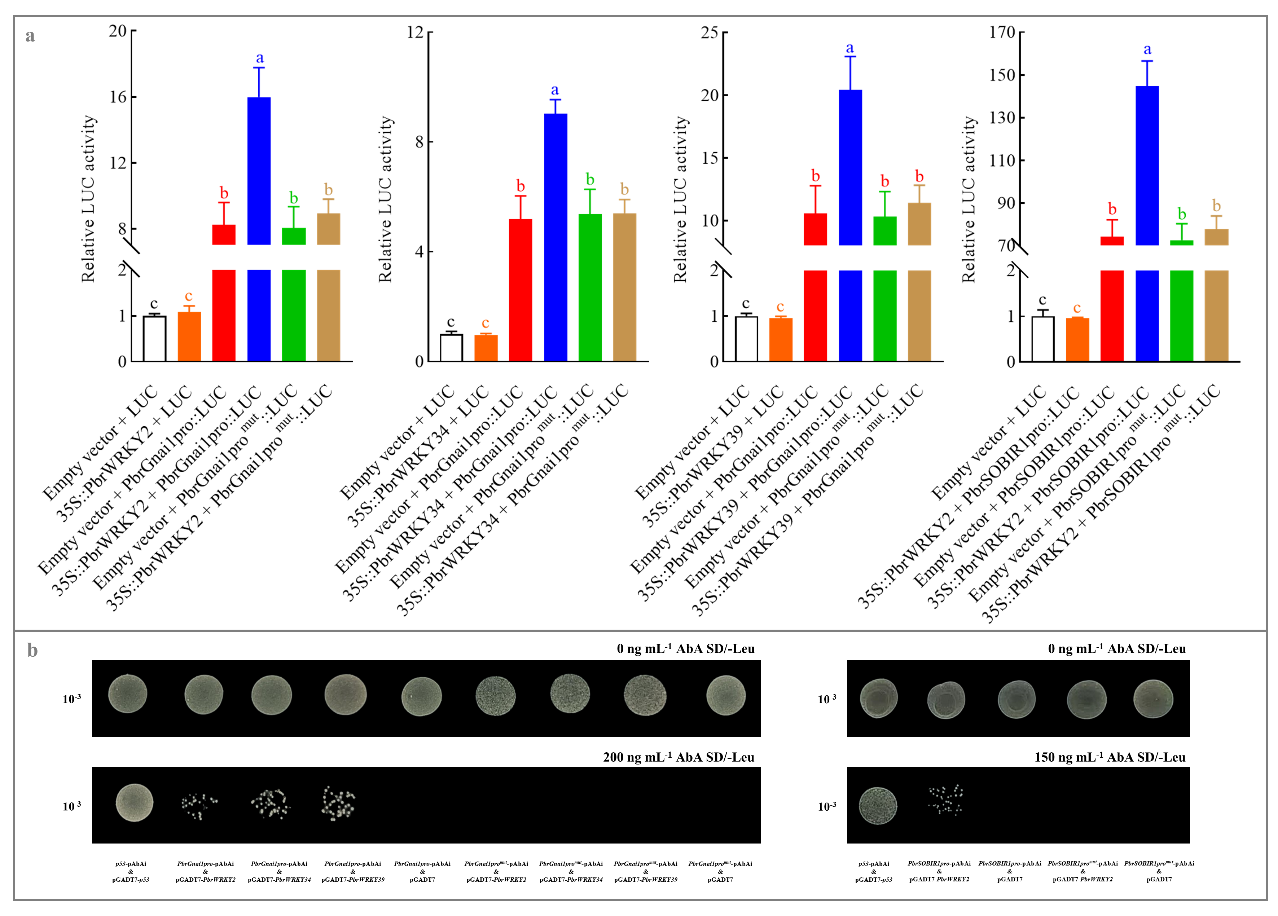


**Fig. S8 Impact of mutation of W-box elements in *PbrGnai1* and *PbrSOBIR1* promoters on their interaction with (or activation by) *PbrWRKY2*, *34*, and *39*. (a) Dual-LUC assay.** Co-transformants containing pCAMBIA1300 & pGreen 0800-LUC vectors, pCAMBIA1300 & pGreen 0800-*PbrGnai1pro*/*PbrSOBIR1pro/PbrGnai1pro^mut^*/*PbrSOBIR1pro^mut^*-LUC vectors, or pCAMBIA1300-*PbrWRKY2/34/39* & pGreen 0800-LUC vectors were used as control. The data are the mean values ± SD of three biological replicates, and vertical bars labelled labeled with the same letter indicate no significant difference between samples at *P <* 0.05. **(b) Y1H assay.** Yeast cell co-transformed with AD-*p53* & *p53*-AbAi was used as the positive control, while yeasts co-transformed with the empty AD vector and each bait as the negative controls


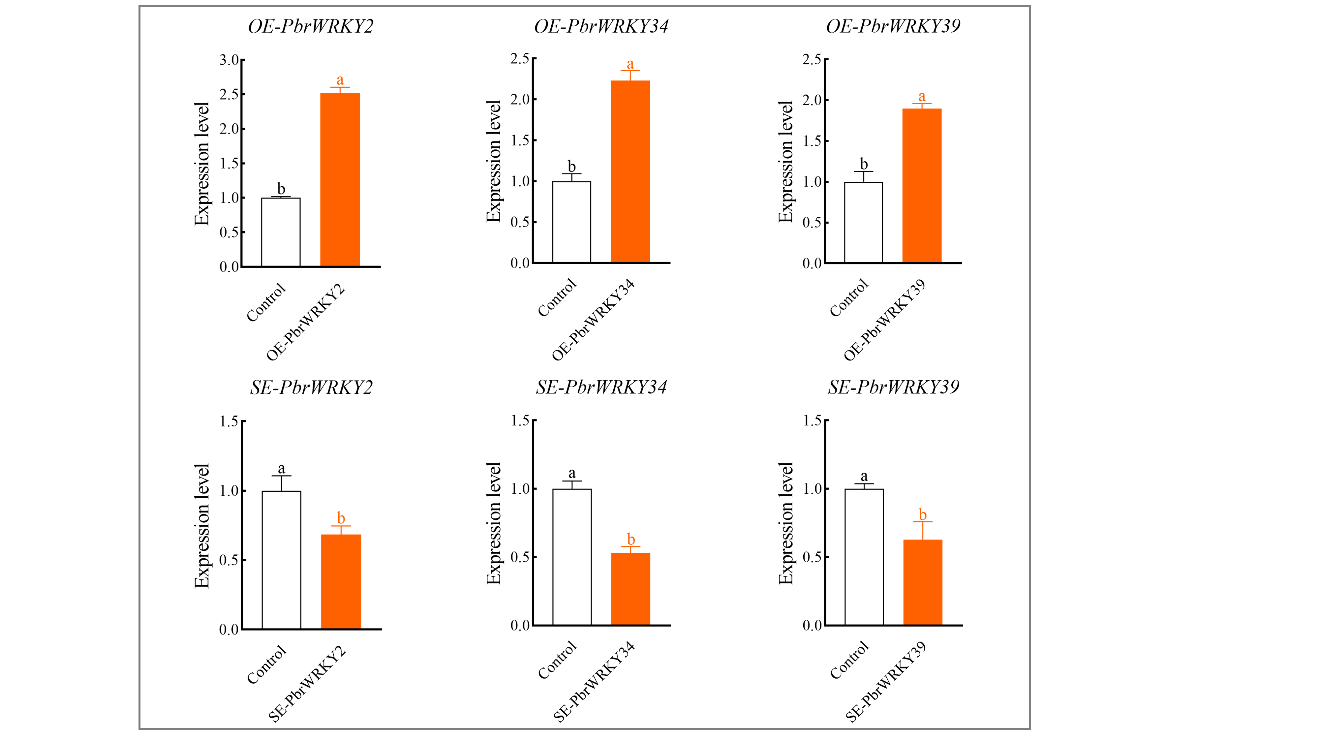


**Fig. S9 Impact of transient transformation of pear fruit on *PbrWRKY2*, *34* and *39* expression abundances.** Fruit transformed with the empty pCAMBIA1300 vector was used as a control for TF-overexpressing fruit, while fruit co-transformed with empty TRV2 and TRV1 was used as a control for TF-silenced fruit. Expression abundance of each gene in control fruit was set as 1.0 based on qRT-PCR result. The data are the mean values ± SD of three biological replicates. Vertical bars labeled with the same letter indicate no significant difference between samples at the same sampling time at *P <* 0.05


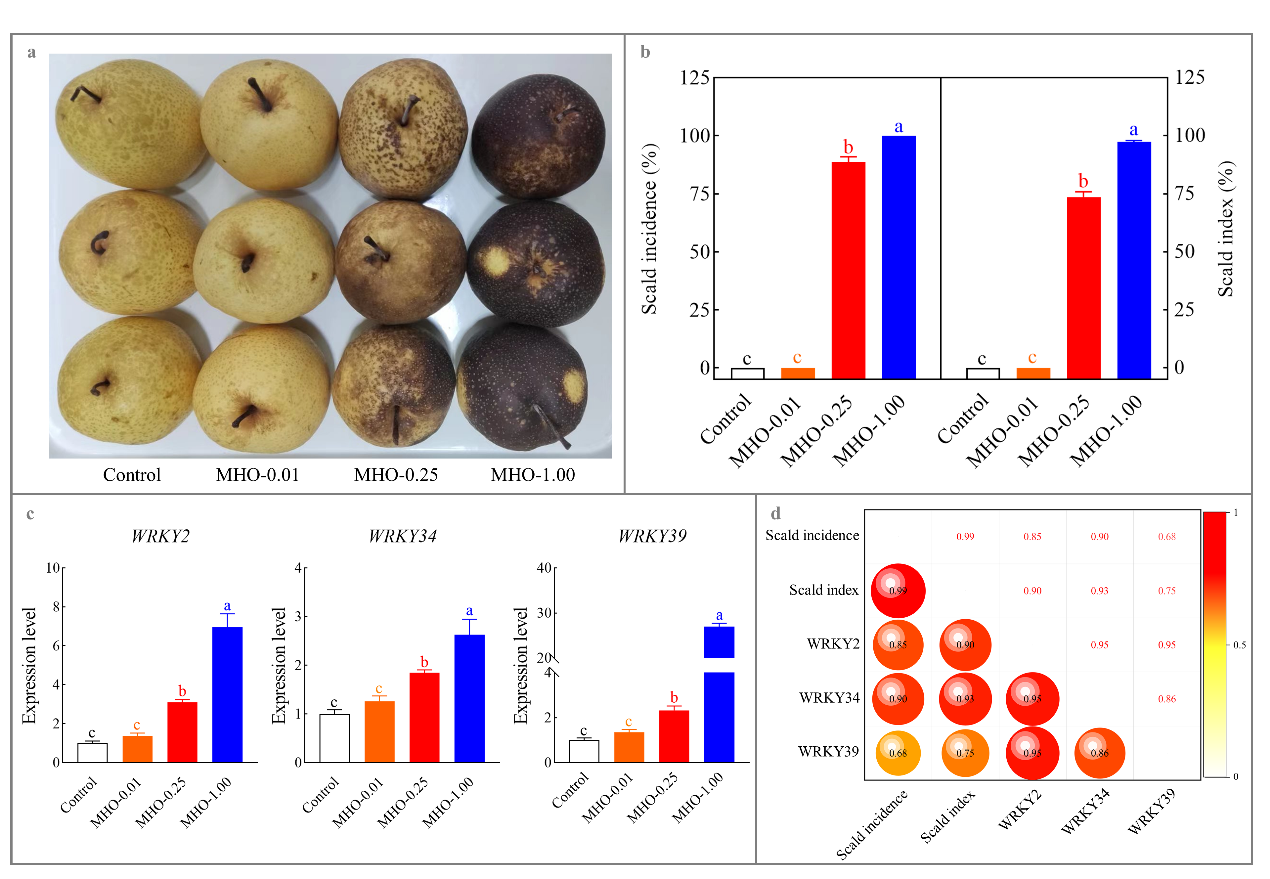


**Fig. S10 Impact of MHO fumigation concentration on scald development and *PbrWRKY2*, *34* and *39* expression levels in ‘Dangshansuli’ fruit. (a) Visual quality. (b) Scald incidence and index. (c) *PbrWRKY2*, *34* and *39* expression levels. (d) Correlations among attributes.** ‘Dangshansuli’ fruits were randomly divided into four groups for the 48-h fumigation with 0.00 (control), 0.01, 0.25, and 1.00 mL L^-1^ MHO, prior to a 7-d shelf life at 20 ℃. Expression abundance of each gene in control fruit was set as 1.0 based on qRT-PCR result. The data are the mean values ± SD of three biological replicates. Vertical bars labeled with the same letter indicate no significant difference between samples at the same sampling time at *P <* 0.05


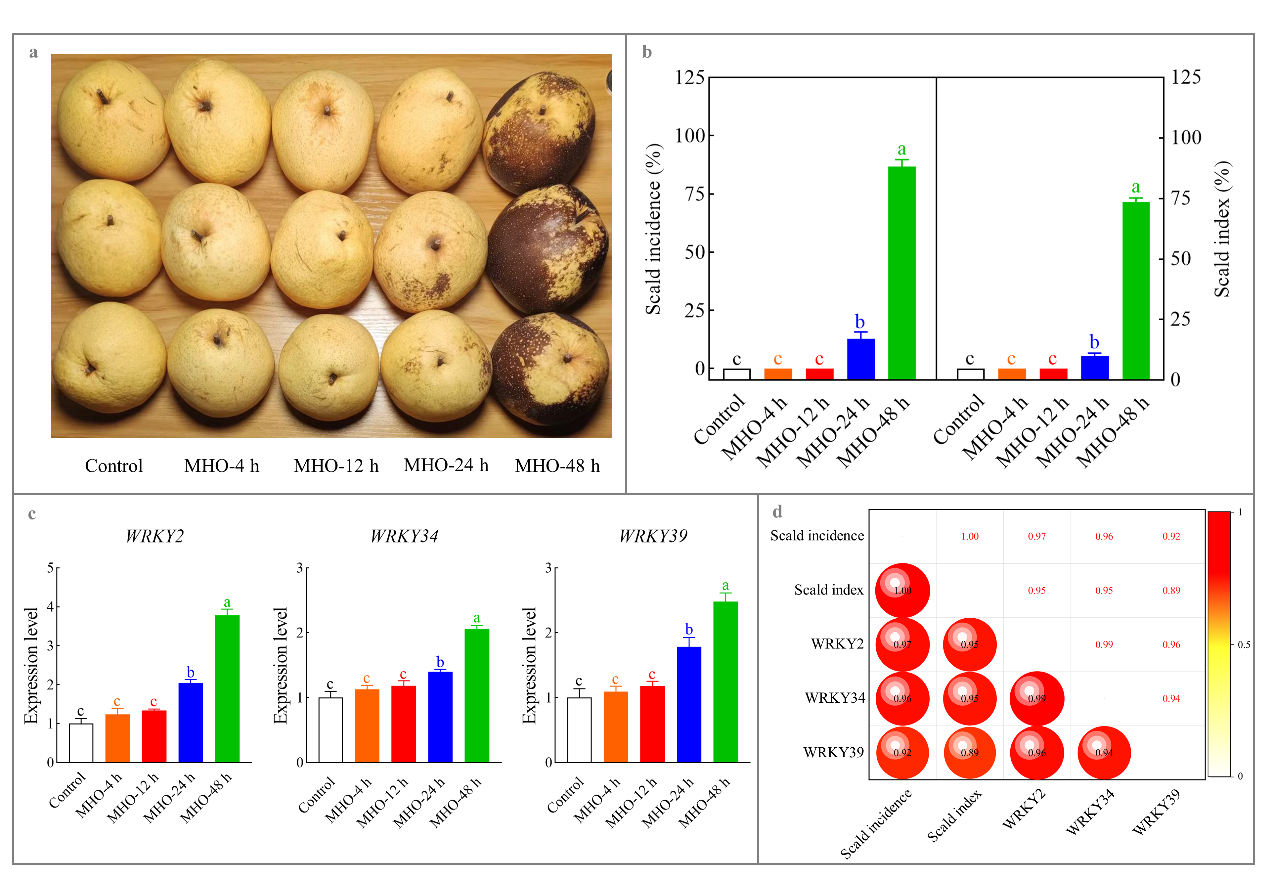


**Fig. S11 Impact of MHO fumigation time on scald development and *PbrWRKY2*, *34* and *39* expression levels in ‘Dangshansuli’ fruit. (a) Visual quality. (b) Scald incidence and index. (c) *PbrWRKY2*, *34* and *39* expression levels. (d) Correlations among attributes.** ‘Dangshansuli’ fruits were randomly divided into five groups for 0.25 mL L^-1^ MHO fumigation for 0 (control), 4, 12, 24, and 48 h, prior to a 7-d shelf life at 20 ℃. Expression abundance of each gene in control fruit was set as 1.0 based on qRT-PCR result. The data are the mean values ± SD of three biological replicates. Vertical bars labeled with the same letter indicate no significant difference between samples at the same sampling time at *P <* 0.05
